# Gata4 is critical to maintain gut barrier function and mucosal integrity following epithelial injury

**DOI:** 10.1038/srep36776

**Published:** 2016-11-09

**Authors:** David Lepage, Élise Bélanger, Christine Jones, Sarah Tremblay, Joannie M. Allaire, Joannie Bruneau, Claude Asselin, Nathalie Perreault, Alfredo Menendez, Fernand-Pierre Gendron, Francois Boudreau

**Affiliations:** 1 Department of Anatomy and Cell Biology, Faculty of Medicine and Health Sciences, Université de Sherbrooke, Quebec, Canada; 2 Department of Microbiology and Infectious Diseases, Faculty of Medicine and Health Sciences, Université de Sherbrooke, Quebec, Canada

## Abstract

The intestinal epithelial barrier is critical to limit potential harmful consequences from exposure to deleterious luminal contents on the organism. Although this barrier is functionally important along the entire gut, specific regional regulatory mechanisms involved in the maintenance of this barrier are poorly defined. Herein, we identified Gata4 as a crucial regulator of barrier integrity in the mouse proximal intestinal epithelium. Conditional deletion of Gata4 in the intestine led to a drastic increase in claudin-2 expression that was associated with an important increase of gut barrier permeability without causing overt spontaneous inflammation. Administration of indomethacin, a non-steroidal anti-inflammatory drug (NSAID) that causes enteritis, led to rapid and restricted proximal small intestinal injuries in Gata4 mutant mice as opposed to control mice. Comparative analysis of gene transcript profiles from indomethacin-challenged control and Gata4 mutant mice identified defects in epithelial cell survival, inflammatory cell recruitment and tissue repair mechanisms. Altogether, these observations identify Gata4 as a novel crucial regulator of the intestinal epithelial barrier and as a critical epithelial transcription factor implicated in the maintenance of proximal intestinal mucosal integrity after injury.

The small intestinal epithelium is a dynamic system that constantly and rapidly regenerates throughout individual life. The continuous production of intestinal epithelial cells is ensured by crypt based columnar stem cells capable of producing progenitors that will differentiate upon distinct integrated molecular programs responsible for the specification of the main cell lineages including enterocytes, goblet, enteroendocrine and Paneth cells^1^. Tight regulation of this process is crucial in order to ensure basic epithelium functions and the integrity of the intestinal barrier that protects against potential harmful luminal content^2^. Toward this end, the intestinal epithelium maintains a permissive mechanical barrier function strictly dependent on apical junctional complexes between epithelial cells that include tight junctions and adherens junctions^3^. Tight junctions localize to the apical end of the lateral surface of adjacent epithelial cells and consist of several transmembrane proteins such as occludin and claudins that mediate adhesion and barrier formation as well as paracellular diffusion^4^. The exact molecular composition and relative expression level of claudins can modulate the overall properties of tight junctions. While most claudins are recognized to maintain strong junctional integrity, several evidences support an opposite role for the claudin-2 member^5^. When overexpressed in Madin-Darby canine kidney (MDCK) epithelial cells, claudin-2 was able to increase tight junction paracellular cation permeability^6^. In addition, knockout mice for *Clnd2* display decreased transepithelial conductance of the small intestinal epithelium^7^. Thus, claudin-2 is functionally associated with tight junction leakiness and its modulation upon biological processes or pathological conditions correlates well with decreased intestinal barrier integrity^8^,^9^.

A subset of transcriptional regulators has been reported to promote intestinal epithelial cell polarization and differentiation. Cdx2 was originally identified as a master regulator of this process in cultured cells^10^. With the use of conditional mouse knockout strategies, Cdx2 has been further established as being crucial for intestinal epithelial cell fate adoption as well as apical-basolateral polarity^11^,^12^. Both Hnf4α and Hnf1α were also identified to modulate cell polarization and differentiation both in culture^13^ and in the mouse intestinal epithelium^14^. Gata4, a member of zinc finger-containing GATA transcription factor family, regulates several intestinal epithelial genes in combination with Cdx2, Hnf4α and Hnf1α^15^,^16^. The expression of Gata4 in the intestine is mostly restricted to the proximal small intestinal epithelium and the generation of *Gata4*-conditional intestinal epithelial knockout mouse models elucidated some of its functions in this context. Indeed, Gata4 regulates specific gene networks along the anterior-caudal axis of the gut epithelium by activating jejunum specific genes^17^,^18^ and by repressing the ileal gene expression program^19^,^20^. Intestinal deletion of *Gata4* also affects lipid metabolism^17^, induces bile acid absorption from the jejunum^21^ and was recently proposed to act as a mediator of the gut microbiota-dependent negative effects on jejunum bile acid absorption^22^. Gata4 was also recently shown to regulate intestinal epithelial expression of regenerating islet derived family members (REG)^23^ for which some members are involved in defense mechanisms and epithelial maintenance of the small intestinal epithelium^24^,^25^.

Here we investigated the potential role for Gata4 in regulating intestinal mucosal barrier integrity. Our findings support a crucial function for this transcriptional regulator in actively repressing the leaky tight junction claudin-2 component, maintaining gut barrier properties and also preventing acute mucosal injury in the proximal small intestine.

## Results

### Gata4 intestinal epithelial deletion impacts functional barrier integrity and regulation of claudin-2 expression

**Figure 1:**
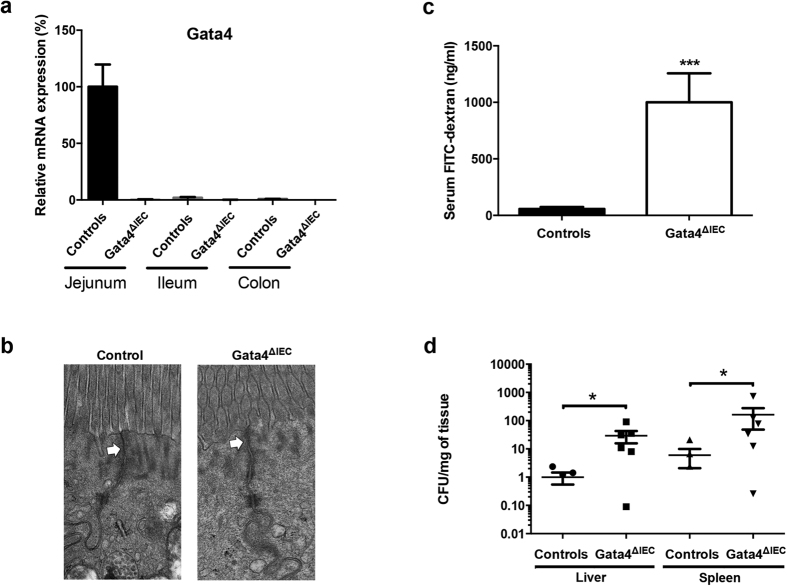
Conditional deletion of *Gata4* in the mouse intestinal epithelium negatively impacts mucosal barrier integrity. (**a**) Total RNA was isolated from the jejunum, ileum and colon of control and *Gata4*^ΔIEC^ mice (n = 3–4 per group) and RT-qPCR was performed to quantify *Gata4* gene transcripts. (**b**) Electron microscopic analysis of jejunum epithelial cells from control and *Gata4*^ΔIEC^ mice. White arrows indicate apical tight junctions. (**c**) Intestinal permeability was assessed by measuring circulating FITC-dextran levels 4 h following gavage of control and *Gata4*^ΔIEC^ mice (n = 8–10 per group). ***P < 0.001 (Student-t test). (**d**) Control and *Gata4*^ΔIEC^ mice were orally infected with *Salmonella typhimurium* SB103 and bacterial counts were done in liver and spleen (n = 5–6 per group). *P < 0.05 (Mann-Whitney test).

*Gata4* intestinal epithelial conditional knockout mice were generated from previously characterized *Gata4*^*loxP*^ allele mice^26^ with *Villin*-Cre transgenic mice that exclusively express Cre in the intestinal epithelium^27^. *Gata4*^ΔIEC^ mice were significantly deleted for *Gata4* gene transcript expression in the jejunum when compared to control littermates (Fig. 1a), while weak expression was recorded in the most distal portion of the ileum and no expression in the colon of control mice, as originally reported^18^. *Gata4* deletion was also confirmed at the level of Gata4 protein expression, as visualized by immunofluorescence (data not shown)^23^. Since the overall expression of Gata4 was substantially more important in the proximal intestine than distally, we focused on the proximal region of the small intestine. Electron microscopy was first performed to monitor the overall integrity of enterocytes in absence of Gata4. Epithelial cells from control mice displayed typical junctional complexes between cells with sealed tight junction structures at the enterocytes apex (Fig. 1b). Although overall epithelial cell ultrastructures were similar among *Gata4*^ΔIEC^ and control mice, tight junctions systematically appeared less well defined in *Gata4*^ΔIEC^ mice when compared to controls (Fig. 1b). Since it was difficult to quantitatively evaluate potential tight junction defects based on ultrastructure analysis, intestinal permeability was next assessed after *Gata4*^ΔIEC^ and control groups of mice were gavaged with FITC-labeled dextran. Detection of FITC concentrations in the serum of *Gata4*^ΔIEC^ mice was significantly increased more than 18.2-fold (*P* < 0.001) when compared to littermate controls (Fig. 1c). To further understand the physiological impact of such increases in mucosal permeability and to determine whether it results in increased translocation of intestinal bacteria, mice were orally infected with the *Salmonella typhimurium* invasion-deficient strain SB103. Due to a mutation in the *invA* gene, this strain is unable to invade enterocytes^28^,^29^ and therefore, its translocation to the mucosa solely depends on other mechanisms (*e*. *g*., increased epithelial permeability). Oral infections of *Gata4*^ΔIEC^ mice with *Salmonella typhimurium* SB103 led to significant increases in liver (29.3-fold; *P* < 0.05) and spleen (26.9-fold; *P* < 0.05) colonization when compared to infected controls (Fig. 1d). Taken together, these observations support the existence of intestinal barrier defects in the absence of Gata4.

**Figure 2:**
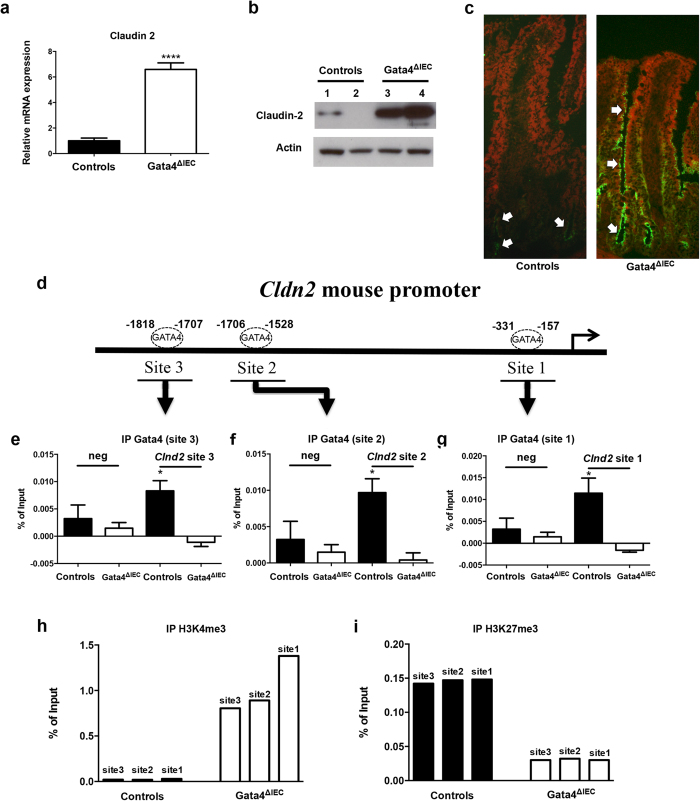
Claudin-2 expression is derepressed in the jejunum of *Gata4* mutant mice. (**a**) Total RNA was isolated from the jejunum of control and *Gata4*^ΔIEC^ mice (n = 3–4 per group) and RT-qPCR was performed to quantify claudin-2 gene transcripts. ****P < 0.0001 (Student-t test). (**b**) Western blot analysis was performed using a claudin-2 polyclonal antibody on total lysates prepared from control and *Gata4*^ΔIEC^ mice. An actin polyclonal antibody was used as a loading control to monitor protein integrity. Cropped blots are displayed and full-length blots are included in the supplementary information. (**c**) Immunofluorescence detection of claudin-2 on jejunum sections prepared from control and *Gata4*^ΔIEC^ mice. Arrows display typical labeling in the tight junctions. Original magnification: 10X. (**d**) Schematic representation of the *Cldn2* gene with its predicted binding sites for GATA. (**e**–**g**) ChIP analysis of three paired biological samples (n = 3) obtained from the jejunum of control and *Gata4*^ΔIEC^ mice. Data were obtained by qPCR and are expressed as the percent of total DNA input used for precipitation with an antibody against Gata4 relative to the DNA precipitated with normal goat-IgG. *P < 0.05 (ANOVA test). (**h**,**i**) ChIP analysis from the jejunum of control and *Gata4*^ΔIEC^ mice. Data were obtained by qPCR and are expressed as the percent of total DNA input used for precipitation with an antibody against H3K4me3 (**h**) or H3K27me3 (**i**) relative to the DNA precipitated with normal IgG.

To explore by which mechanisms Gata4 could transcriptionally influence epithelial permeability, gene transcript expression of several molecules functionally involved in the formation of junctional complexes were quantified in *Gata4*^ΔIEC^ and control mouse jejunum samples (Supplementary Table 1). From this analysis, claudin-2 was found to be the most drastically modulated transcript with a significant 6.6-fold increase (*P* < 0.0001) in *Gata4*^ΔIEC^ mice in comparison to controls (Fig. 2a). This induction of expression was also reflected at the protein level as determined by Western blot on total jejunum extracts (Fig. 2b) and immunofluorescence on jejunum sections (Fig. 2c). Interestingly, claudin-2 was strongly detected at the apex of crypt and differentiated enterocytes in *Gata4*^ΔIEC^ mice while mostly restricted to crypt epithelial cells in control mice (Fig. 2c). Gata4 was previously identified to positively regulate transcription of the human *CLDN2* gene promoter^30^. Sequence analysis of the 5′-flanking region of the murine *Clnd2* gene predicted three putative GATA elements for which one was located in the TSS vicinity of the promoter (Fig. 2d). ChIP experiments were then conducted on mouse jejunum isolated from control and *Gata4*^ΔIEC^ mice. Antibody specific for Gata4 was able to precipitate mouse wild-type jejunum chromatin encompassing the three predicted GATA-binding sites of the murine *Cldn2* gene but failed to precipitate mouse *Gata4*^ΔIEC^ jejunum chromatin under the same conditions (Fig. 2e–g). To further monitor whether loss of Gata4 interaction with the *Cldn2* gene could be functionally linked to active chromatin, histone modification methylation marks of the *Cldn2* gene were quantified in the presence or absence of Gata4. Pull-down of H3K4me3, that labels active chromatin, showed a drastic enrichment of mouse *Gata4*^ΔIEC^ jejunum chromatin in the vicinity of *Cldn2* GATA interacting sites (Fig. 2h). Coincidentally, H3K27me3, a repressive histone modification marker showed a decreased association with *Gata4*^ΔIEC^ jejunum chromatin surrounding *Cldn2* GATA binding sites (Fig. 2i). Taken together, these observations support a direct role for Gata4 in repressing *Cldn2* gene transcription in mouse jejunum epithelial cells.

### Impaired intestinal mucosal barrier integrity in *Gata4* ^ΔIEC^ mice does not lead to spontaneous intestinal inflammation

**Figure 3:**
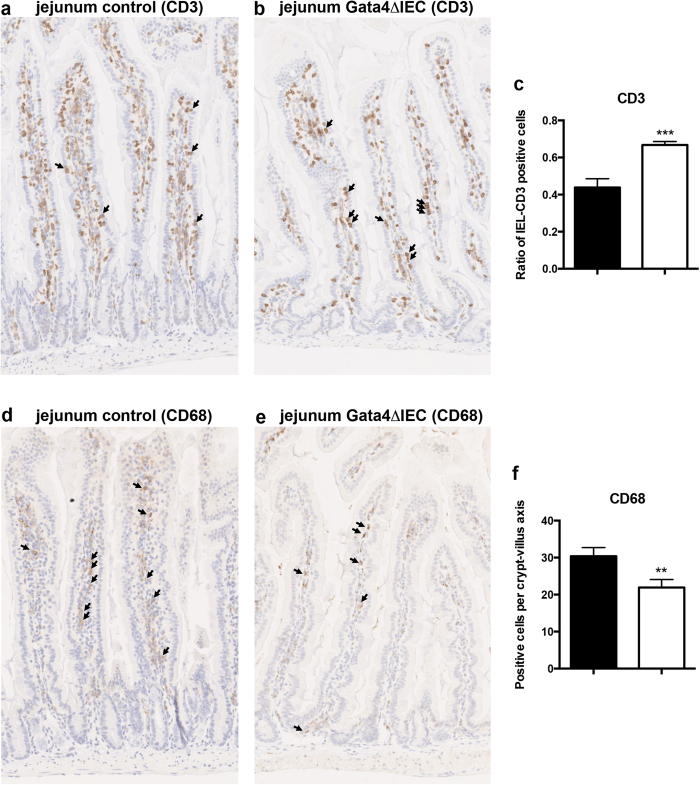
Immune cells detection in the jejunum of *Gata4* mutant mice. T lymphocytes were visualized by immunohistochemistry against CD3 in control (**a**) and *Gata4*^ΔIEC^ (**b**) mice. (**c**) The ratio of intra-epithelial lymphocytes (IEL) per total number of CD3 positive cells was averaged from 5 different crypt-villus axes of a total of 3 different mice per group. ***P < 0.001 (Student-t test). Macrophages were visualized by immunohistochemistry against CD68 in control (**d**) and *Gata4*^ΔIEC^ (**e**) mice. (**f**) The total number of CD68 positive cells was averaged from 5 different crypt-villus axes of a total of 3 different mice per group. **P < 0.01 (Student-t test). Original magnification 10X.

Histological observations of the small intestine from *Gata4*^ΔIEC^ mice did not reveal signs of inflammation as late as 1 year of age (Fig. 3a,b). A mouse inflammatory response qPCR array performed on jejunum extracts did not indicate important inflammation related changes when *Gata4*^ΔIEC^ mice were compared to controls (Supplementary Table 2). A careful examination of the jejunum distribution of inflammatory cells was next compared between *Gata4*^ΔIEC^ and control mice. While no significant modification in the total number of CD3 positive cells per villus was noted between *Gata4*^ΔIEC^ and control mice, a modest but significant increase in the ratio of CD3-positive intraepithelial lymphocytes (IELs) (1.4 fold; *P* < 0.0001) was observed in the mucosa of *Gata4*^ΔIEC^ mice when compared to controls (Fig. 3a–c). A modest but significant decrease in the number of CD68-labeled macrophages per villus (1.5 fold; *P* < 0.001) was also observed in the jejunum of *Gata4*^ΔIEC^ mice when compared to controls (Fig. 3d,e). These observations suggest that increased barrier permeability in non-inflammatory challenged *Gata4*^ΔIEC^ mice modestly affected their jejunum mucosal immunity and do not spontaneously lead to intestinal inflammation.

### *Gata4* ^ΔIEC^ mice are severely sensitive to indomethacin-induced small intestinal injury response

**Figure 4:**
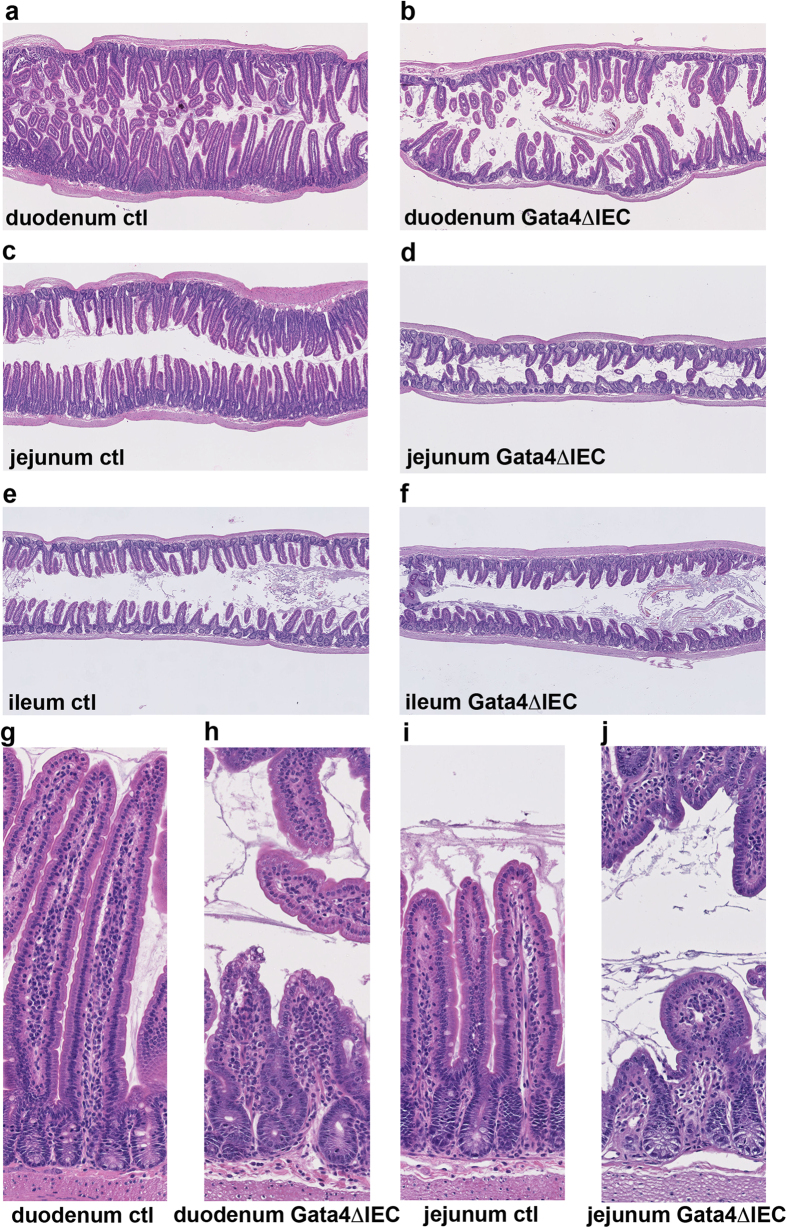
Indomethacin severely impairs proximal small intestinal integrity of Gata4 mutant mice. Control (**a**,**c**,**e**,**g**,**i**) and *Gata4*^ΔIEC^ (**b**,**d**,**f**,**h**,**j**) mice were sacrificed 24 h after indomethacin injection and sections from the duodenum (**a**,**b**,**g**,**h**), the jejunum (**c**,**d**,**i**,**j**) and the ileum (**e**,**f**) were stained with H&E. Images are representative of at least 3 individuals per group. Original magnification: 2.5X (**a**–**f**) and 10X (**g**–**j**).

Indomethacin-induced small intestinal inflammation model was next used to investigate whether absence of jejunum Gata4 expression could sensitize mice to injury insults. Control and *Gata4*^ΔIEC^ mice were injected with a single dose of indomethacin (10 mg/kg) and rapidly sacrificed 24 h after treatment due to the severity of *Gata4*^ΔIEC^ mice response. Histological assessment of the small intestinal mucosa following this treatment revealed drastic villi regression in the duodenum of *Gata4*^ΔIEC^ mice (Fig. 4b,h) as opposed to controls (Fig. 4a,g). This effect was also observed in the jejunum of *Gata4*^ΔIEC^ mice (Fig. 4d,j) when compared to controls (Fig. 4c,i). Interestingly, ileum histology was similar between *Gata4*^ΔIEC^ (Fig. 4f) and control (Fig. 4e) treated groups. We next investigated whether these changes in intestinal epithelium integrity can relate to proliferation defects of progenitor crypt epithelial cells. PCNA labeling on intestinal sections of *Gata4*^ΔIEC^ and control indomethacin-treated mice was relatively constant among the crypts of both control (Supplementary Fig. 1a) and *Gata4*^ΔIEC^ (Supplementary Fig. 1b) treated mice, without an overall significant change in the ratio of labeled cells per number of crypt cells (Supplementary Fig. 1c). This observation suggests that indomethacin-induced small intestinal mucosal injuries in absence of Gata4 were not originating from alterations of the epithelial cell proliferative pools but rather defects in epithelial cell viability or adherence.

**Figure 5:**
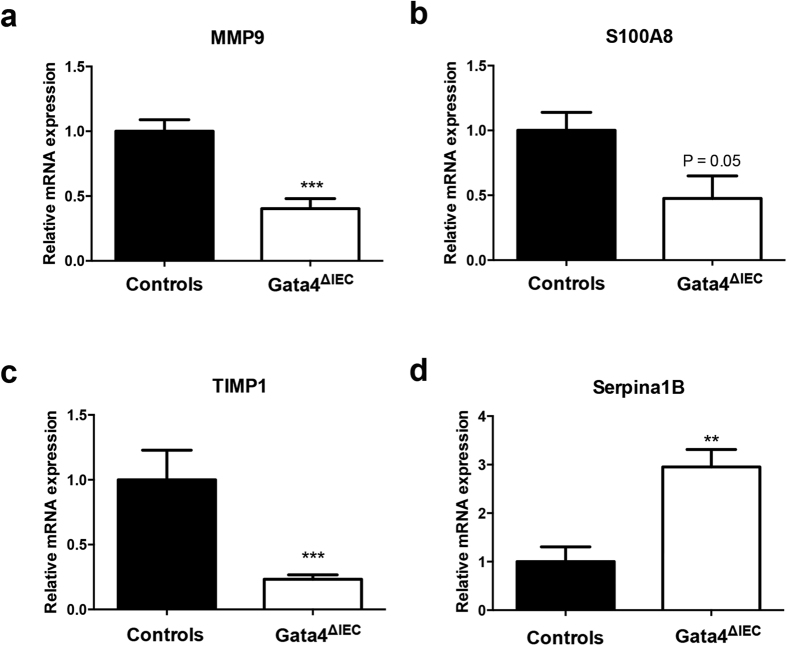
Expression of inflammatory modulators after indomethacin treatment of Gata4 mutant mice. Total RNA was isolated from the jejunum of control and *Gata4*^ΔIEC^ mice that were sacrificed after indomethacin injection (n = 4–10 per group). RT-qPCR was performed to quantify MMP9 (**a**), S100A8 (**b**), TIMP1 (**c**) and Serpina1B (**d**) gene transcripts. **P < 0.01; ***P < 0.001 (Student-t test).

**Figure 6:**
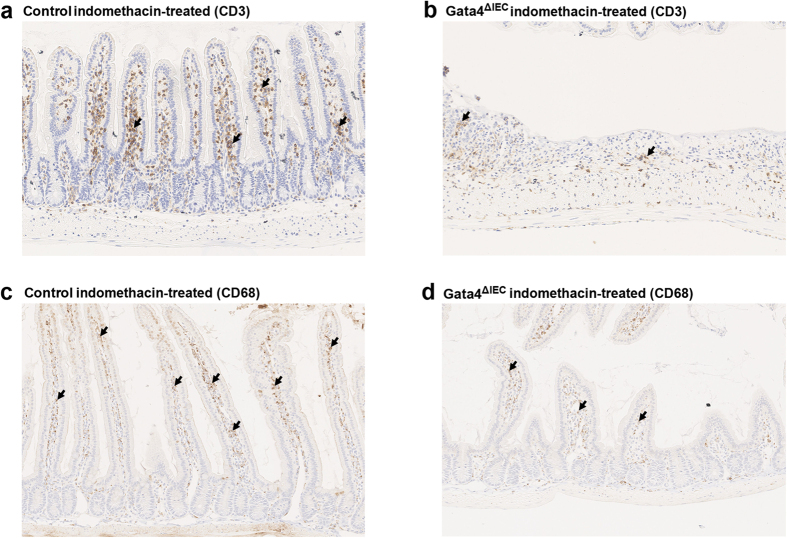
Macrophages and T-lymphocytes mucosal distribution after indomethacin treatment of Gata4 mutant mice. T lymphocytes were visualized by immunohistochemistry against CD3 in control (**a**) and *Gata4*^ΔIEC^ (**b**) indomethacin-treated mice. Macrophages were visualized by immunohistochemistry against CD68 in control (**c**) and *Gata4*^ΔIEC^ (**d**) indomethacin-treated mice (black arrows). The images were representative of 3 different mice per group. Original magnification: 10X.

In order to clarify the nature of the signals linked with increased mucosal injury in the absence of Gata4, a gene expression profiling was next performed. This analysis was done in biological triplicate with RNA isolated from the jejunum of indomethacin-treated control and indomethacin-treated *Gata4*^ΔIEC^ mice. The Illumina Mouse WG-6 v2.0 Expression Bead chip that contains more than 45,200 transcripts from the mouse genome was utilized to screen for mRNA expression variations. A statistical analysis (*P* value ≤0.05) predicted 309 unique and mapped transcripts being significantly modulated between control and *Gata4*^ΔIEC^ indomethacin-treated mice (differential ratio ≥2.0; Supplementary Table 3). To gain insight into how these modifications could be classified as biological meaning, we used the Ingenuity Pathway Analysis (IPA) software. This analysis identified the haematological system and cellular movement as being the top network functions affected in the *Gata4*^ΔIEC^ indomethacin-treated mice. Disorders in nutrition (40 molecules; *P* value range between 8E-03 and 5E-09) and in inflammatory response (64 molecules; *P* value range between 9E-03 and 3E-07) were identified as the top affected diseases and biological functions. Death of epithelial cells was also predicted to be increased (26 molecules; *P* value of 8E-06) (Supplementary Fig. 2) while immune cell trafficking (34 molecules; *P* value of 2E-04) was predicted to be decreased (Supplementary Fig. 3). These predicted changes in expression were next assessed for some candidate genes clustered among these networks of biological functions. A qRT-PCR analysis confirmed that matrix metallopeptidase 9 (MMP9) (2.4-fold reduction; *P* < 0.001) (Fig. 5a), S100 calcium binding protein A8 (S100A8) (2.1-fold reduction; *P* = 0.05) (Fig. 5b), tissue inhibitor of metalloproteinase 1 (TIMP1) (4.7-fold reduction; *P* < 0.001) (Fig. 5c) and Serpina1B (3.4-fold induction; *P* < 0.01) (Fig. 5d) gene transcripts were significantly modulated in the jejunum of *Gata4*^ΔIEC^ indomethacin-treated mice when compared to control indomethacin-treated mice. In accordance to the predicted decrease in blood cells movement, the number of CD3-positive T cells was lower in the jejunum of *Gata4*^ΔIEC^ indomethacin-treated mice (Fig. 6b) when compared to control treated mice (Fig. 6a). A similar observation was made for CD68-positive macrophages (Fig. 6c,d).

## Discussion

The intestinal epithelial barrier integrity is critical to prevent the translocation of luminal components to the mucosa and to maintain the physiological and immunological homeostasis of the gut. In homeostatic conditions, pathogenic or environmental insults to the intestinal epithelium would stimulate the mucosal immune system and not necessarily lead to disease^31^,^32^. However, a leaky gut barrier might both facilitate some level of constitutive inflammation and exacerbates the pro-inflammatory effects of the insults, given the increased exposure of the immune system to intruding luminal content that includes intestinal bacteria and their products. Our observations show that even though Gata4 deficiency results in a compromised intestinal epithelial barrier, the small intestine remains free of overt inflammation. At first glance, this could appear contradictory. Indeed, several genetically engineered mouse models for which gut epithelial barrier function has been altered displayed defects in their mucosal immune response^33^,^34^,^35^. However, one commonality among those models is that amplified immune response was mainly localized to the gut distal part. One possible explanation for these observations is the significant lower bacterial load in the jejunum because of higher antimicrobial activities when compared to the gut distal part^36^. This could explain why Gata4 mutant mice lack significant spontaneous inflammatory symptoms in the absence of nutritional or environmental stresses similar to what is observed for specific proximal gut disorders such as celiac disease^37^.

The defect of barrier integrity as observed in the Gata4 mutant mice is mechanistically linked to the direct interaction between Gata4 and *Clnd2* transcription in the jejunum. Claudin-2 is well accepted to act by itself as a mediator of leaky gut barrier during intestinal inflammation^9^ as well as during exposure to microbial products^38^. Gata4 was previously shown to stimulate transcription of a *Clnd2* promoter construct under artificial co-transfection assays in cultured cells^30^. In contrast, our data indicate that deletion of Gata4 leads to the activation of *Cldn2* promoter in the jejunum and a subsequent increase in claudin-2 expression. Gata4 can act either as an activator or a repressor of gene transcription, depending on the nature of contextually-recruited co-regulators. Friend of Gata-1 (Fog-1) acts as a co-repressor of Gata4 in the small intestine^20^ and negatively regulates the expression of several genes including some members of the REG family^23^. Whether Fog1 is functionally involved during Gata4 dependent repression of *Clnd2* remains to be investigated, but our observations support a novel and global repressive role for Gata4 in claudin-2 expression and barrier integrity in the context of an *in vivo* physiological system. How the increase in claudin-2 expression promotes mucosal permeability to FITC-dextran and *Salmonella* in Gata4 mutant mice remains unclear. Claudin-2 forms a channel that is selectively permeable to small cations but not for molecules such as FITC-dextran or pathogens^9^. Whether the increase in claudin-2 expression alters other tight junction components or modifies the pattern of tight junction strands of intestinal epithelial cells in Gata4 mutants are possible. Since no reduction in tight junction gene transcripts expression was observed in the jejunum of Gata4 mutants and since ZO-1 protein distribution remains similar under these conditions (Supplementary Fig. 4), we postulate that the increase of claudin-2 and resulting changes in combination and mixing ratios of claudin molecules could influence the overall tightness of tight junction strands^39^. In addition, increased gut permeability in these mice could also be caused by apoptosis resulting in epithelial cells loss.

NSAIDs are well described to cause damages to the human gastrointestinal tract but the exact mechanisms involved are not yet completely understood^40^. Indomethacin treatment in mice often results in damage restricted to the more distal regions of the small intestine and will manifest after a relatively long period of time. Our findings that Gata4 mutant mice become rapidly and highly sensitive to the deleterious effects of indomethacin in the proximal small intestine are, to our knowledge, unique and provide the opportunity to investigate in more detail the overall nature of the mechanisms involved during NSAID induced mucosal injuries. One interesting clue as to how Gata4 might be involved in protecting the jejunum from such effects relates to the NSAID’s potential to preferentially cause enteropathy by combination with bile^41^ and changes in the gut microbiota^42^. Intriguingly, bile acid absorption is induced in the jejunum of Gata4 mutant mice^21^ and gut microbiota can inhibit bile acid reabsorption through Gata4^22^.

Although Gata4 mutant mice did not show important spontaneous inflammation, their jejunum displayed minor but significant increases in IELs content. IELs are composed of a mixed population of lymphocytes and are believed to play an important role in protecting the epithelial barrier^43^. Increase of their mucosal recruitment to a leaky barrier would make sense in this context. However, our data indicate that the jejunum of Gata4 mutant mice is more prone to tissue damage following indomethacin treatment. It is tempting to speculate that subclasses of activated IELs might participate into the initial steps of deleterious damaging effects caused by indomethacin as it is observed in the case of celiac disease^43^. However, the transcriptome analysis coupled to the immunolocalization of immune cells in the jejunum of short-term indomethacin-treated Gata4 mutant mice supports that the recruitment of immune cells and the inflammatory response are inhibited under these conditions. S100A8, abundantly expressed in immune cells of myeloid origin and thought to be functionally involved during epithelial wound healing of several tissues^44^,^45^, was found to be decreased in injured mutant mice. MMP-9, an endopeptidase involved in wound healing through regulating the turnover of matrix proteins^46^, was also found decreased under these conditions. It is intriguing that MMP-9 was found to be increased during the healing of indomethacin-induced small intestinal damage in rats and that administration of MMP inhibitors significantly impaired the healing of ulceration during this treatment^47^. Serpina1, described as a potent blocker of hematopoietic stem cell mobilization^48^, was found to be increased in injured mutant mice. It is also plausible that the jejunal epithelium of Gata4 mutant mice be intrinsically less competent for epithelial restitution. In support of this, we recently reported that Reg1, a crucial factor for the maintenance of the villous structure of the small intestine^25^, was spontaneously reduced in the jejunum of Gata4 mutant mice^23^.

Our study identifies Gata4 as an epithelial transcriptional regulator crucial for the maintenance of physiological barrier integrity, as well as for the protection of the jejunal mucosa against epithelial injury. Few model systems are currently available to define the epithelial intrinsic contribution involved in proximal gut protection against inflammatory stimuli. For instance, specific transgenic mouse models with modified MHC class II molecules have been generated and showed to recapitulate some aspects of celiac disease pathogenesis when exposed to dietary gluten^43^. Upon our analyses, Gata4 deleted mice did not display classical signs of celiac disease associated signature, such as for instance, modification in the expression of IL-15 or activating natural killer receptor NKG2D gene transcripts (data not shown). Our findings open up on exploring whether Gata4 and its regulatory network are involved in the pathogenesis and/or the protection against environmental damage and inflammatory diseases of the proximal gastrointestinal tract.

## Methods

### Animals

*Gata4*^*loxP*^^26^ and 12.4Kb*Vil*Cre^27^ mice were used to generate 12.4Kb*Vil*Cre/*Gata4*^+/+^ (control) and 12.4Kb*Vil*Cre/*Gata4*^*loxP/loxP*^ (*Gata4*^ΔIEC^) mice on a pure C57BL/6J background. Mice were kept under pathogen free conditions and were tested negative for *Helicobacter*, *Pasteurella* and murine norovirus. Some of the mice were injected a single dose of indomethacin (Sigma-Aldrich Canada Co., Oakville, ON) (10 mg/kg body weight) intraperitoneally and based on the observed severity phenotype for mutant mice, sacrificed 24 h later for tissue samples. Mice were treated in accordance with a protocol reviewed and approved by the Institutional Animal Research Review Committee of the Université de Sherbrooke (approval ID number 102-10B). The study followed the standards and policies of the Canadian Council on Animal Care in sciences.

### RNA isolation and qPCR analysis

Total RNA was isolated from jejunum, ileum and colon mouse biopsies and subjected to a DNase treatment according to the manufacturer’s instructions (Totally RNA kit, Life Technologies Inc., Burlington, ON). Reverse transcription and quantitative PCR (qPCR) were performed as described previously^14^,^49^ or were performed by the RNomics Platform at the Université de Sherbrooke (Sherbrooke, QC). Target expression was quantified relatively to porphobilinogen deaminase (PBGD) expression. Primer sequences used for qPCR are listed in Supplementary Table S4.

### Electron microscopy

Mouse jejunum segments were prepared as reported before^49^. Ultramicrotome-prepared thin sections were contrasted with lead citrate and uranyl acetate and then observed on a Jeol 100 CX transmission electron microscope.

### Intestinal permeability *in vivo*

Permeability was assessed with the fluorescent isothiocyanate (FITC)-labeled dextran method as described previously^50^. Mice were oral gavage with 60 mg/100g body weight of FITC-dextran (FD4, average molecular weight of 3,000–5,000, Sigma-Aldrich Canada Co., Oakville, ON) and sacrificed after 4 h. FITC concentration in the serum was quantified with a BioTek Synergy HT spectrometer plate reader (Winooski, VT) with excitation of the fluorophore at 492 nm and emission at 525 nm. Serum from mice not administered with FITC-dextran was used to determine the background.

### Bacterial strain and mouse infections

*Salmonella enterica* serovar Typhimurium strain SB103 (*invA*) was grown overnight at 37 °C in LB supplemented with 100 μg/mL streptomycin. Inoculum was prepared in sterile HEPES 100 mM, NaCl 0.9%, pH 8.0. Mice were infected orally with 5 × 10^7^ bacteria as previously described^29^, and sacrificed after 3 days. For bacterial counts, tissues were homogenized, followed by plating of serial dilutions in LB plates containing 100 μg/mL streptomycin. All infections experiments were done in duplicate with a total of 5–6 mice per group.

### Immunostaining

Immunofluorescence and immunohistochemistry staining was performed as previously described^51^. Non-specific binding was blocked and antibodies were diluted in PBS/Triton 0.05% solution containing 2% BSA (Sigma-Aldrich Canada Co., Oakville, ON). The following antibodies were used at the indicated dilutions: anti-PCNA (1:1000, Abcam), anti-CD3 (1:200, Dako), anti-CD68 (1:500, Aviva), FITC-conjugated anti-rabbit IgG (1:300, Santa Cruz), Alexa 568-conjugated anti-mouse (1:400, Invitrogen). For claudin-2 and ZO-1 immunodetection, 5 μm thick OCT cryosections were fixed in 100% methanol for 10 min at −20 °C and further processed for incubations with antibodies. Immunohistochemistry staining (DAB kit, Dako) was performed following the manufacturer’s protocol.

### Immunoblot analysis

Total protein extracts and western blots were performed as described previously^51^. The following antibodies were used: anti-claudin-2 (#51-6100, 1:500) (Invitrogen, Life Technologies Inc., Burlington, ON) and anti-β-actin (MAB1501R, 1:10,000) (EMD Millipore, Etobicoke, ON).

### Chromatin immunoprecipitation assays

Chromatin immunoprecipitation assays (ChIP) were performed with the EZ-ChIP assay kit (EMD Millipore, Etobicoke, ON), according to the manufacturer’s instructions. The jejunum was harvested from mice, cut opened in fragments and incubated in 1% formaldehyde for 15 min at 20 °C. Jejunum fragments were then rinsed twice with ice cold PBS/Glycine buffer and mucosal isolation was performed by scraping. Enriched mucosal fractions were weighed and a total of 40 mg was used for each condition. Mucosal fractions were chemically crosslinked by the addition of formaldehyde (1% final concentration) for 10 min at 20 °C, then lysed and sonicated to solubilize and shear crosslinked DNA to an average length of 200 base pairs. The cell extract was pre-cleared with 40 μl Protein G magnetic beads (Life Technologies Inc, Burlington, ON) for 1 h and then, incubated at 4 °C overnight with 40 μl Protein G magnetic beads and corresponding antibodies: 4 μg of an isogenic immunoglobulin (Santa Cruz Biotechnology Inc., Santa Cruz, CA), 4 μg of GATA-4 affinity-purified polyclonal antibody (#SC-1237, Santa Cruz Biotechnology Inc., Santa Cruz, CA), 4 μg of anti-Histone H3 (tri methyl K4) purified polyclonal antibody (#ab8580, Abcam, Toronto, ON) or 4 μg of anti-trimethyl-Histone H3 (Lys27) purified polyclonal antibody (#07-449, EMD Millipore, Etobicoke, ON). Beads were washed twice with the provided buffers. Bound complexes were eluted from the beads by heating at 65 °C with occasional vortexing, and crosslinking was reversed by overnight incubation at 65 °C. A portion of the whole-cell extract DNA from the sonication step was also treated for crosslink reversal for input. Immunoprecipitated DNA and whole-cell extract DNA (1% input) were treated with proteinase K and RNaseA and then purified. Purified DNA was used as template for qPCR with a LightCycler apparatus V2.0 (Roche Diagnostics, Laval, QC). Sets of primers used to amplify GATA containing regions for *Cldn2* gene promoter and for a negative region from the *Il1β* gene promoter are listed in Supplementary Table S5. Calculation of enrichment was performed using the 2-ΔΔCt method that normalized ChIP DNA to input DNA and included signals obtained from both wild-type and *Gata4*^ΔIEC^ mouse jejunum extracts^23^.

### DNA microarray and analysis

Probes for hybridization with Illumina BeadChips were generated from isolated jejunum RNA of three independent mice from both *Gata4*^ΔIEC^ and control groups after 24 h of indomethacin treatment. The Illumina MouseWG-6 v2.0 Expression BeadChips were screened with the six generated probes via the McGill University and Génome Québec Innovation Center ( http://genomequebec.mcgill.ca). FlexArray version 1.6.1 was used for data analysis ( http://genomequebec.mcgill.ca/FlexArray). Genes were then filtered for up- or down-regulation of expression of a minimum of 2.0-fold and gene signature datasets analyzed by the Ingenuity Pathway Analysis tool ( www.ingenuity.com).

## Additional Information

**How to cite this article**: Lepage, D. *et al.* Gata4 is critical to maintain gut barrier function and mucosal integrity following epithelial injury. *Sci. Rep.* **6**, 36776; doi: 10.1038/srep36776 (2016).

**Publisher’s note:** Springer Nature remains neutral with regard to jurisdictional claims in published maps and institutional affiliations.

## Supplementary Material

Supplementary Information
