# Supplementary material for: Gata4 is critical to maintain gut barrier function and mucosal integrity following epithelial injury
Source: Sci Rep. 2016 Nov 9;6:36776. doi: 10.1038/srep36776 (PMC5101531; doi:10.1038/srep36776)
Supplement: Supplementary Information [file srep36776-s1.pdf]

Supplementary information

Gata4 is critical to maintain gut barrier function and mucosal integrity following epithelial injury

David Lepage, Élise Bélanger, Christine Jones, Sarah Tremblay, Joannie Allaire, Joannie Bruneau, Claude Asselin, Nathalie Perreault, Alfredo Menendez, Fernand-Pierre Gendron and Francois Boudreau

Supplementary Figure legends

Figure S1. Indomethacin does not affect jejunum epithelial cell proliferation in Gata4 mutant mice. PCNA positive cells were detected by immunofluorescence on the jejunum of control (**a**) and *Gata4*$^{\Delta IEC}$ (**b**) mice that were sacrificed 24 h after indomethacin injection. Original magnification: 2.5X. (**c**) The ratio of PCNA-positive cells was expressed in percentage of total number of crypt cells and was averaged from 8 individual crypts of a total of 3 different mice per group.

Figure S2. Transcriptomic analysis predicts increased epithelial cell death in the jejunum of Gata4 mutant mice following indomethacin treatment. Total RNA was isolated from the jejunum of control and *Gata4*$^{\Delta IEC}$ mice that were sacrificed after indomethacin injection (n=3 per group) and probes for hybridization with Illumina BeadChips were generated. Genes were then filtered for up- or down-regulation of expression of a minimum of 2.0-fold with a P value ≤ 0.05 and gene signature datasets were analyzed by the Ingenuity Pathway Analysis tool. (**a**) Categories and functions annotations were associated with a p-value, activation state and number of different

molecules found to be modulated in these categories. (**b**) 26 molecules were found to be modulated (up in red and down in green) and associated with predicted increases of epithelial cell death. The color quantification scale of dotted lines associated with biological functions covers between orange (increased state) and blue (decreased state).

Figure S3. Transcriptomic analysis predicts decreased immune cell trafficking in the jejunum of Gata4 mutant mice following indomethacin treatment. Total RNA was isolated from the jejunum of control and *Gata4*$^{\Delta IEC}$ mice that were sacrificed after indomethacin injection (n=3 per group) and probes for hybridization with Illumina BeadChips were generated. Genes were then filtered for up- or down-regulation of expression of a minimum of 2.0-fold with a *P* value ≤ 0.05 and gene signature datasets were analyzed by the Ingenuity Pathway Analysis tool. (**a**) Categories and functions annotations were associated with a p-value, activation state and number of different molecules found to be modulated in these categories. (**b**) 34 molecules were found to be modulated (up in red and down in green) and associated with predicted decreases of immune cell trafficking. The color quantification scale of dotted lines associated with biological functions covers between orange (increased state) and blue (decreased state).

Figure S4. ZO-1 localization remains unchanged in the jejunum of *Gata4* mutant mice. Immunofluorescence detection of ZO-1 on jejunum sections prepared from controls and *Gata4*$^{\Delta IEC}$ mice. Original magnification: 10X.

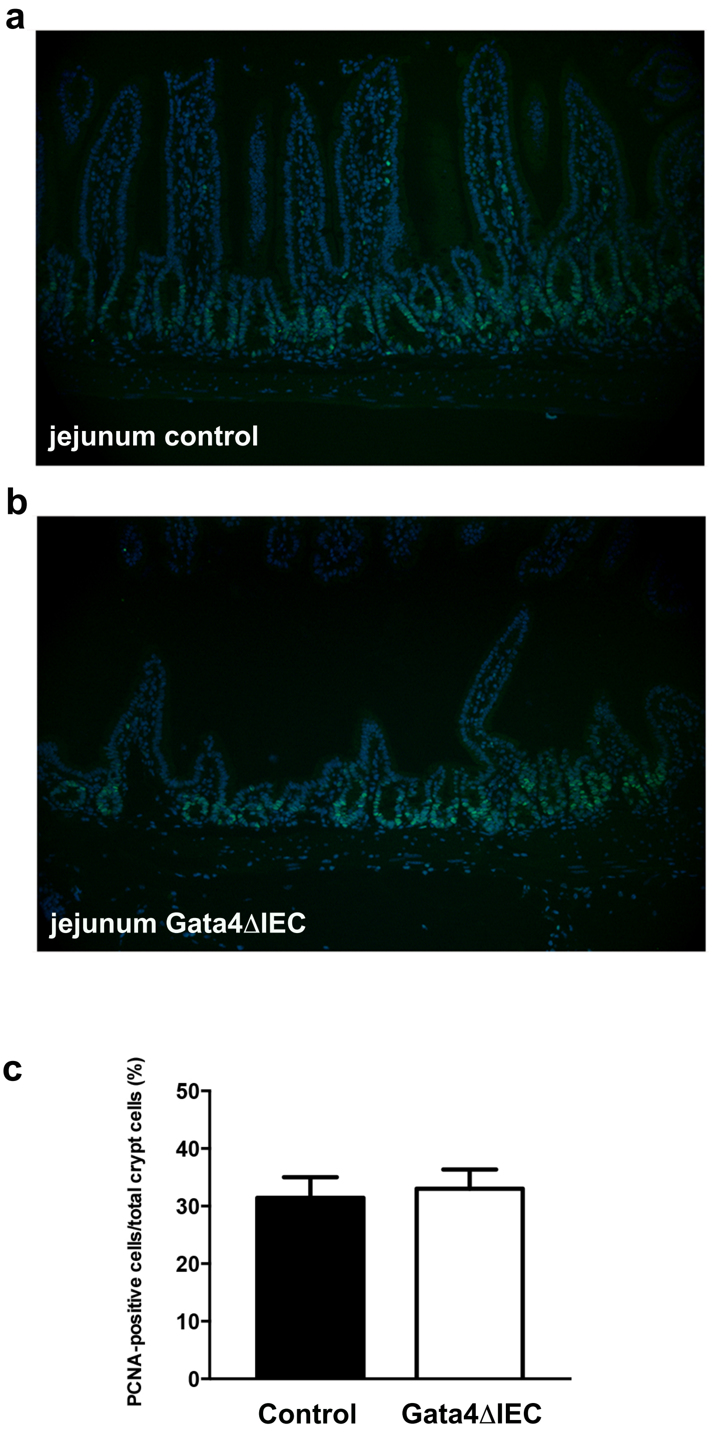

Figure S1. Lepage et al.

A

Categories	Diseases or Functions Annotation	p-Value	Predicted Activation State	Activation z-score	# Molecules
Cell Death and Survival	necrosis	1,86E-07		1,664	93
Cell Death and Survival, Gastrointestinal Disease, Hepatic System Disease	cell death of hepatocytes	5,66E-07	Increased	2,251	14
Cell Death and Survival	cell death	7,76E-07		1,479	111
Cell Death and Survival, Gastrointestinal Disease, Hepatic System Disease	cell death of liver cells	2,68E-06		1,691	15
Cell Death and Survival, Respiratory Disease	apoptosis of lung cells	6,61E-06		0,896	7
Cell Death and Survival	cell death of epithelial cells	7,60E-06	Increased	2,233	26
Cell Death and Survival, Gastrointestinal Disease, Hepatic System Disease	necrosis of liver	1,06E-05		1,488	16
Cell Death and Survival	necrosis of epithelial tissue	3,00E-05		1,954	28
Cell Death and Survival	apoptosis	3,14E-05		1,594	86
Cell Death and Survival, Gastrointestinal Disease, Hepatic System Disease	apoptosis of hepatocytes	9,71E-05		1,659	10
Cell Death and Survival, Gastrointestinal Disease, Hepatic System Disease	apoptosis of liver cells	9,74E-05		1,052	11
Cell Death and Survival	cell death of enteroendocrine cells	1,44E-04			3
Cell Death and Survival	necrosis of kidney	2,41E-04		1,497	18
Cell Death and Survival	apoptosis of connective tissue cells	3,04E-04		0,375	15
Cell Death and Survival	apoptosis of epithelial cells	3,57E-04		1,392	15
Cell Death and Survival	cell death of kidney cells	3,96E-04		1,332	17
Cell Death and Survival, Respiratory Disease	cell death of pneumocytes	4,04E-04		1,982	4
Cell Death and Survival	cell death of tumor cell lines	6,02E-04		1,561	51
Cell Death and Survival	cell death of splenocytes	6,51E-04		0,447	5
Cell Death and Survival, Cellular Compromise, Reproductive System Development	cytotoxicity of gonadal cell lines	7,89E-04			2
Cell Death and Survival, Respiratory Disease	quantity of apoptotic pneumocytes	7,89E-04			2
Cell Death and Survival	cell death of colon	1,09E-03			3
Cell Death and Survival	apoptosis of tumor cell lines	1,16E-03		1,702	41
Cell Death and Survival	cell death of striatal neurons	1,31E-03		0,468	5
Cell Death and Survival, Embryonic Development	apoptosis of embryonic cell lines	1,32E-03	Increased	2,043	10
Cell Death and Survival, Embryonic Development	cell death of embryonic cell lines	1,49E-03		1,965	12
Cell Death and Survival	cell death of endocrine cells	1,51E-03		1,461	7
Cell Death and Survival	delay in initiation of removal of cells	1,56E-03			2
Cell Death and Survival	necrosis of colon	1,56E-03			2
Cell Death and Survival	cell death of kidney cell lines	1,66E-03		1,595	14
Cell Death and Survival, Respiratory Disease	apoptosis of pneumocytes	2,06E-03			3
Cell Death and Survival	opsonization	2,47E-03			3
Cell Death and Survival	cell death of islet cells	3,19E-03		1,461	6
Cell Death and Survival	quantity of apoptotic cells	3,57E-03		0,6	6
Cell Death and Survival	apoptosis of kidney cell lines	3,90E-03		1,336	11
Cell Death and Survival	cell death of pericytes	6,00E-03			3
Cell Death and Survival	apoptosis of uterine cell lines	6,97E-03			2
Cell Death and Survival	opsonization of cells	6,97E-03			2
Cell Death and Survival	cell death of intestinal cells	8,30E-03			4

B

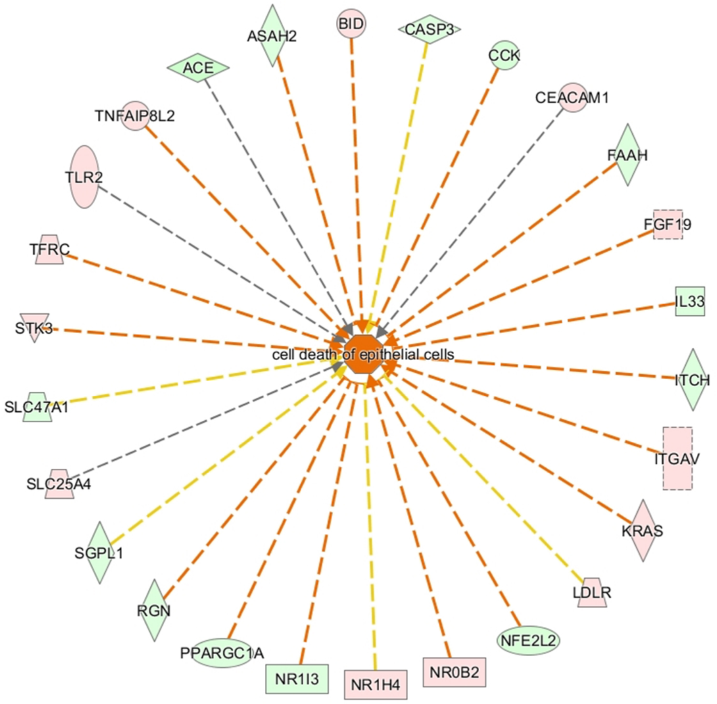

Figure S2. Lepage et al.

A

Categories	Diseases or Functions Annotation	p-Value	Predicted Activation State	Activation	# Molecules
Cellular Movement, Hematological System Development and Function, Immune Cell Trafficking	cell movement of myeloid cells	1,66E-04	Decreased	-2,183	20
Cellular Movement, Hematological System Development and Function, Immune Cell Trafficking	influx of leukocytes	1,93E-04		-0,816	6
Hematological System Development and Function, Immune Cell Trafficking, Inflammatory Response, Tissue Development	accumulation of neutrophils	2,44E-04		-1,777	6
Cellular Movement, Hematological System Development and Function, Immune Cell Trafficking, Inflammatory Response	cell movement of neutrophils	2,57E-04		-1,576	13
Cellular Movement, Hematological System Development and Function, Immune Cell Trafficking, Inflammatory Response	migration of neutrophils	2,75E-04		-0,656	7
Cell-To-Cell Signaling and Interaction, Hematological System Development and Function, Immune Cell Trafficking, Inflammatory Response,	adhesion of neutrophils	2,82E-04		-0,883	6
Hematological System Development and Function, Immune Cell Trafficking, Inflammatory Response, Tissue Development	accumulation of phagocytes	4,44E-04		-1,671	8
Cellular Movement, Hematological System Development and Function, Immune Cell Trafficking, Inflammatory Response	cell movement of phagocytes	5,89E-04		-1,834	19
Cellular Movement, Hematological System Development and Function, Immune Cell Trafficking	infiltration of granulocytes	6,06E-04		-1,795	10
Cellular Movement, Hematological System Development and Function, Immune Cell Trafficking, Inflammatory Response	chemotaxis of myeloid cells	7,85E-04		-1,874	12
Cellular Movement, Hematological System Development and Function, Immune Cell Trafficking, Inflammatory Response	chemotaxis of phagocytes	8,69E-04		-1,835	12
Cellular Movement, Hematological System Development and Function, Immune Cell Trafficking	cell movement of granulocytes	9,29E-04		-1,695	14
Cellular Movement, Hematological System Development and Function, Immune Cell Trafficking	homing of leukocytes	9,92E-04		-1,271	14
Cell-To-Cell Signaling and Interaction, Hematological System Development and Function, Immune Cell Trafficking, Inflammatory Response	activation of myeloid cells	1,04E-03		-1,087	13
Cellular Movement, Hematological System Development and Function, Immune Cell Trafficking	migration of myeloid cells	1,06E-03		-0,701	8
Cellular Movement, Immune Cell Trafficking	leukocyte migration	1,10E-03	Decreased	-2,35	26
Cellular Movement, Hematological System Development and Function, Immune Cell Trafficking, Inflammatory Response	infiltration by neutrophils	1,27E-03	Decreased	-2,155	8
Cellular Movement, Hematological System Development and Function, Immune Cell Trafficking, Inflammatory Response	chemotaxis of neutrophils	1,37E-03		-1,466	8
Cellular Movement, Hematological System Development and Function, Immune Cell Trafficking, Inflammatory Response	chemotaxis of leukocytes	1,55E-03		-1,879	13
Cellular Movement, Hematological System Development and Function, Immune Cell Trafficking	infiltration of leukocytes	1,60E-03	Decreased	-2,047	14
Cellular Movement, Hematological System Development and Function, Immune Cell Trafficking	cell movement of leukocytes	1,65E-03		-1,735	23
Hematological System Development and Function, Immune Cell Trafficking, Inflammatory Response, Tissue Development	accumulation of myeloid cells	1,87E-03		-1,687	8
Cellular Movement, Hematological System Development and Function, Immune Cell Trafficking, Inflammatory Response	chemokinesis of neutrophils	2,89E-03			2
Cell-To-Cell Signaling and Interaction, Hematological System Development and Function, Immune Cell Trafficking, Inflammatory Response	activation of neutrophils	4,58E-03		-1,949	5
Cell-To-Cell Signaling and Interaction, Hematological System Development and Function, Immune Cell Trafficking, Inflammatory Response	activation of leukocytes	4,59E-03		-0,996	20
Cellular Movement, Hematological System Development and Function, Immune Cell Trafficking, Inflammatory Response	migration of phagocytes	4,89E-03		-0,636	10
Cell-To-Cell Signaling and Interaction, Hematological System Development and Function, Immune Cell Trafficking, Inflammatory Response	activation of phagocytes	5,53E-03		-0,84	12
Cell-To-Cell Signaling and Interaction, Cellular Movement, Hematological System Development and Function, Immune Cell Trafficking	recruitment of granulocytes	7,09E-03	Decreased	-2,244	8
Cell-To-Cell Signaling and Interaction, Hematological System Development and Function, Immune Cell Trafficking, Inflammatory Response	activation of peripheral blood neutrophils	1,20E-02			1
Cellular Movement, Hematological System Development and Function, Immune Cell Trafficking, Inflammatory Response	chemokinesis of monocytes	1,20E-02			1

B

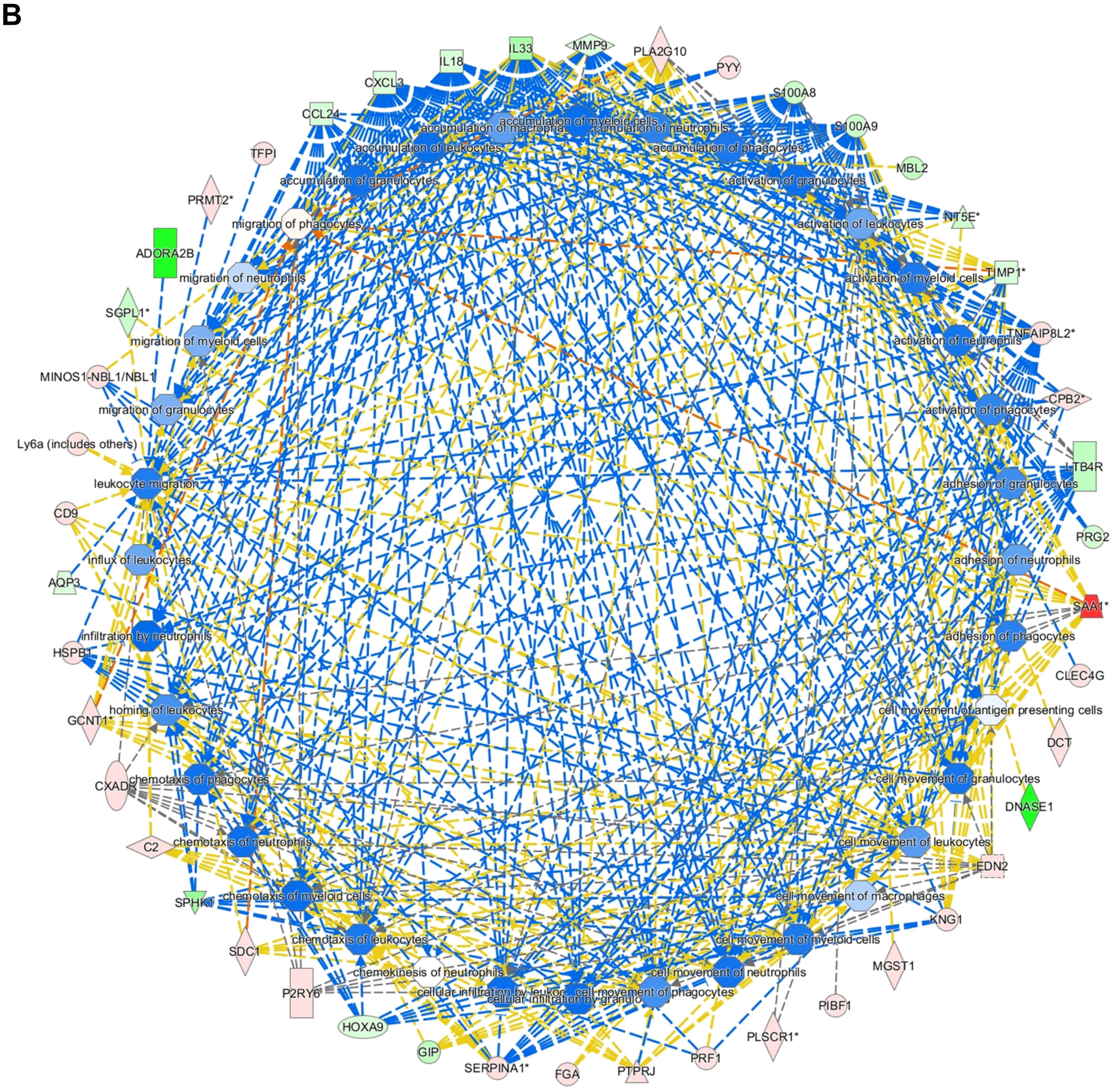

Figure S3. Lepage et al.

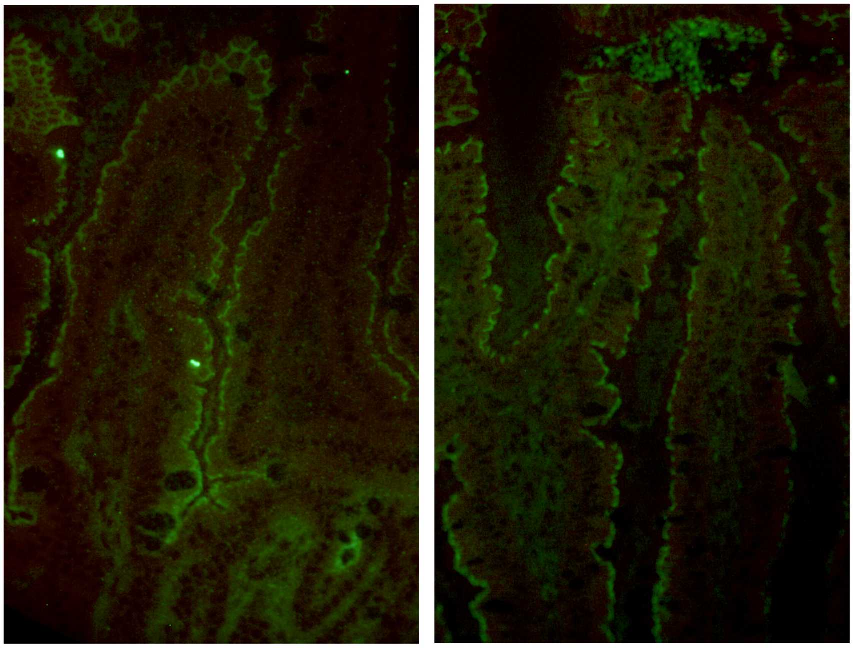

Figure S4. Lepage et al.

Table S1. Comparative gene transcript expression of junctional molecules in the jejunum of *Gata4*$^{\Delta IEC}$ and control mice (n=6 for each group).

Gene transcript	Fold difference (*Gata4*$^{\Delta IEC}$ /control)	*P* value (Student T-test)
Claudin-1	1.17 ± 0.1	0.16
**Claudin-2**	**6.59 ± 0.52**	**0.0001**
Claudin-3	1.56 ± 0.06	0.0006
ZO-1	1.26 ± 0.07	0.01
JAM-1	1.28 ± 0.08	0.05
Occludin	1.24 ± 0.04	0.02
E-Cadherin	1.45 ± 0.08	0.007

Table S2. Comparative gene transcript expression of inflammatory molecules in the jejunum of *Gata4*$^{\Delta IEC}$ and control mice (n=6 for each group).

Gene transcript	Fold difference (*Gata4*$^{\Delta IEC}$ /control)	*P* value (Student T-test)
Icam1	1.43 ± 0.12	0.02
Cx3cr1	1.55 ± 0.26	0.05
Il-1β	0.62 ± 0.04	0.08
Cd5	1.67 ± 0.52	0.30
Cd69	1.54 ± 0.34	0.19
Il-1βr	1.15 ± 0.10	0.54
Il-23a	0.64 ± 0.13	0.22
Ccl20	1.19 ± 0.44	0.74
Ccr6	1.06 ± 0.55	0.92
Cxcl1	1.08 ± 0.22	0.82
Il-21	0.46 ± 0.15	0.29
Ccr4	0.46 ± 0.15	0.20
Cd25	1.27 ± 0.26	0.36
Cxcr4	1.39 ± 0.43	0.43
Foxp3	0.79 ± 0.26	0.51
Inf-γ	1.57 ± 0.41	0.33
Il-10	1.11 ± 0.14	0.60
Il-12p35	0.63 ± 0.22	0.60
Il-12p40	1.38 ± 0.22	0.42
Il-13	0.02 ± 0.01	0.15
Il-17	1.72 ± 0.90	0.54
Il-2	0.87 ± 0.21	0.67
Il-23r	1.73 ± 0.38	0.12
Il-24	0.67 ± 0.08	0.17
Tgfβ	1.11 ± 0.16	0.61
Tnfα	1.63 ± 0.34	0.15

© 2000-2014 Ingenuity Systems, Inc. All rights reserved.

Fold Change	p-value	ID	Symbol	Entrez Gene Name	Location	Type(s)
-13,036	3,79E-06	Adora2b	ADORA2B	adenosine A2b receptor	Plasma Membrane	G-protein coupled receptor
-9,473	4,35E-05	Slc2a2	SLC2A2	solute carrier family 2 (facilitated glucose transporter), member 2	Plasma Membrane	transporter
-9,335	5,74E-05	Slc28a1	SLC28A1	solute carrier family 28 (concentrative nucleoside transporter), member 1	Plasma Membrane	transporter
-8,888	1,53E-04	Gm766	Gm766	predicted gene 766	Other	other
-6,223	5,30E-05	Apoc2	APOC2	apolipoprotein C-II	Extracellular Space	transporter
-5,883	4,92E-05	Sphk1	SPHK1	sphingosine kinase 1	Cytoplasm	kinase
-5,658	1,96E-05	Bst1	BST1	bone marrow stromal cell antigen 1	Plasma Membrane	enzyme
-5,334	6,88E-05	Apoc2	APOC2	apolipoprotein C-II	Extracellular Space	transporter
-5,301	3,22E-05	Slc25a45	SLC25A45	solute carrier family 25, member 45	Cytoplasm	transporter
-5,234	4,86E-05	Enpp7	ENPP7	ectonucleotide pyrophosphatase/phosphodiesterase 7	Plasma Membrane	enzyme
-5,020	7,89E-05	Acot12	ACOT12	acyl-CoA thioesterase 12	Cytoplasm	enzyme
-4,871	9,15E-05	Car2	CA2	carbonic anhydrase II	Cytoplasm	enzyme
-4,841	8,83E-06	Rec8	REC8	REC8 meiotic recombination protein	Nucleus	other
-4,723	3,47E-05	Enpp7	ENPP7	ectonucleotide pyrophosphatase/phosphodiesterase 7	Plasma Membrane	enzyme
-4,645	2,62E-04	Apoa4	APOA4	apolipoprotein A-IV	Extracellular Space	transporter
-4,552	1,01E-05	Trim38	TRIM38	tripartite motif containing 38	Other	other
-4,503	6,63E-05	Rbp2	RBP2	retinol binding protein 2, cellular	Cytoplasm	transporter
-4,107	2,78E-04	Slc2a2	SLC2A2	solute carrier family 2 (facilitated glucose transporter), member 2	Plasma Membrane	transporter
-4,047	6,12E-05	Rbp2	RBP2	retinol binding protein 2, cellular	Cytoplasm	transporter
-3,824	2,54E-02	Asb11	ASB11	ankyrin repeat and SOCS box containing 11	Nucleus	transcription regulator
-3,809	2,76E-03	Fam151a	FAM151A	family with sequence similarity 151, member A	Extracellular Space	other
-3,777	6,40E-04	Capn13	CAPN13	calpain 13	Other	other
-3,731	2,90E-05	Ltb4r1	LTB4R	leukotriene B4 receptor	Plasma Membrane	G-protein coupled receptor
-3,611	4,73E-05	Trim38	TRIM38	tripartite motif containing 38	Other	other
-3,435	3,52E-03	Fam151a	FAM151A	family with sequence similarity 151, member A	Extracellular Space	other
-3,378	4,90E-04	Adh1	ADH1C	alcohol dehydrogenase 1C (class I), gamma polypeptide	Cytoplasm	enzyme
-3,235	4,83E-04	Cyp2b23	Cyp2b23	cytochrome P450, family 2, subfamily b, polypeptide 23	Other	enzyme
-3,233	2,47E-04	Adh1	ADH1C	alcohol dehydrogenase 1C (class I), gamma polypeptide	Cytoplasm	enzyme
-3,220	2,95E-04	Slc16a6	SLC16A6	solute carrier family 16, member 6	Plasma Membrane	transporter
-3,188	1,78E-04	Tlcd2	TLCD2	TLC domain containing 2	Other	other
-3,181	7,12E-04	Pcsk6	PCSK6	proprotein convertase subtilisin/kexin type 6	Extracellular Space	peptidase
-3,160	6,25E-05	Sec14l2	SEC14L2	SEC14-like 2 (S. cerevisiae)	Cytoplasm	transporter
-3,156	1,83E-03	S100a8	S100A8	S100 calcium binding protein A8	Cytoplasm	other
-3,127	5,09E-04	Slc2a2	SLC2A2	solute carrier family 2 (facilitated glucose transporter), member 2	Plasma Membrane	transporter
-3,095	8,96E-05	Slc1a3	SLC1A3	solute carrier family 1 (glial high affinity glutamate transporter), member 3	Plasma Membrane	transporter
-3,081	4,64E-04	S100a9	S100A9	S100 calcium binding protein A9	Cytoplasm	other
-3,002	1,70E-03	Bdh2	BDH2	3-hydroxybutyrate dehydrogenase, type 2	Cytoplasm	enzyme
-2,991	3,49E-04	Gltpd2	GLTPD2	glycolipid transfer protein domain containing 2	Other	other
-2,952	5,55E-05	Elmo2	ELMO2	engulfment and cell motility 2	Cytoplasm	other
-2,931	1,08E-03	Apoa4	APOA4	apolipoprotein A-IV	Extracellular Space	transporter
-2,929	8,77E-05	Ltb4r2	LTB4R2	leukotriene B4 receptor 2	Plasma Membrane	G-protein coupled receptor
-2,890	4,02E-04	Nt5e	NT5E	5'-nucleotidase, ecto (CD73)	Plasma Membrane	phosphatase
-2,878	1,34E-04	Cda	CDA	cytidine deaminase	Nucleus	enzyme
-2,839	8,20E-05	Lmcd1	LMCD1	LIM and cysteine-rich domains 1	Cytoplasm	transcription regulator
-2,823	9,26E-04	Gm156	Gm156	predicted gene 156	Other	other
-2,821	2,21E-04	Ccdc114	CCDC114	coiled-coil domain containing 114	Extracellular Space	other
-2,810	1,30E-02	Cyp2c37	CYP2C18	cytochrome P450, family 2, subfamily C, polypeptide 18	Cytoplasm	enzyme
-2,795	3,91E-05	Prss12	PRSS12	protease, serine, 12 (neurotrypsin, motopsin)	Extracellular Space	peptidase
-2,783	8,01E-05	Plekhf1	PLEKHF1	pleckstrin homology domain containing, family F (with FYVE domain) member 1	Cytoplasm	other
-2,762	8,14E-05	Lmcd1	LMCD1	LIM and cysteine-rich domains 1	Cytoplasm	transcription regulator
-2,719	2,04E-03	Cyp2d26	Cyp2d26	cytochrome P450, family 2, subfamily d, polypeptide 26	Cytoplasm	enzyme
-2,711	4,03E-04	Tlcd2	TLCD2	TLC domain containing 2	Other	other
-2,686	1,01E-03	Tcrg-V5	Tcrg-V5	T cell receptor gamma, variable 5	Other	other
-2,686	6,18E-05	Vsx1	VSX1	visual system homeobox 1	Nucleus	transcription regulator
-2,684	1,12E-04	Unc93a	UNC93A	unc-93 homolog A (C. elegans)	Plasma Membrane	other
-2,680	1,43E-03	Apoa4	APOA4	apolipoprotein A-IV	Extracellular Space	transporter
-2,661	1,96E-04	Creb3l3	CREB3L3	cAMP responsive element binding protein 3-like 3	Other	other
-2,650	7,19E-05	Ociad2	OCIAD2	OCIA domain containing 2	Cytoplasm	other
-2,642	5,54E-04	2210407C18Rik	2210407C18Rik	RIKEN cDNA 2210407C18 gene	Other	other
-2,627	8,23E-04	Gpr151	GPR151	G protein-coupled receptor 151	Plasma Membrane	G-protein coupled receptor
-2,603	8,33E-05	Ckmt1	Ckmt1	creatine kinase, mitochondrial 1, ubiquitous	Cytoplasm	kinase
-2,603	1,41E-03	Slc16a6	SLC16A6	solute carrier family 16, member 6	Plasma Membrane	transporter
-2,596	7,26E-05	Gde1	GDE1	glycerophosphodiester phosphodiesterase 1	Other	enzyme
-2,592	8,71E-05	Prg2	PRG2	proteoglycan 2, bone marrow (natural killer cell activator, eosinophil granule major basic protein)	Extracellular Space	other
-2,579	1,51E-04	Tnfrsf22	Tnfrsf22/Tnfrsf23	tumor necrosis factor receptor superfamily, member 22	Plasma Membrane	transmembrane receptor
-2,523	1,02E-04	Gde1	GDE1	glycerophosphodiester phosphodiesterase 1	Other	enzyme
-2,498	7,51E-05	Ociad2	OCIAD2	OCIA domain containing 2	Cytoplasm	other
-2,485	1,61E-04	Ttll2	TTLL2	tubulin tyrosine ligase-like family, member 2	Other	other
-2,481	1,40E-04	Mdh2	MDH2	malate dehydrogenase 2, NAD (mitochondrial)	Cytoplasm	enzyme
-2,468	9,52E-03	Cyp2d26	Cyp2d26	cytochrome P450, family 2, subfamily d, polypeptide 26	Cytoplasm	enzyme
-2,456	1,11E-04	Lypd1	LYPD1	LY6/PLAUR domain containing 1	Plasma Membrane	G-protein coupled receptor
-2,453	1,04E-02	Cyp2d26	Cyp2d26	cytochrome P450, family 2, subfamily d, polypeptide 26	Cytoplasm	enzyme
-2,441	3,96E-04	Nt5e	NT5E	5'-nucleotidase, ecto (CD73)	Plasma Membrane	phosphatase
-2,411	1,55E-04	Paqr7	PAQR7	progestin and adipoQ receptor family member VII	Plasma Membrane	other
-2,407	2,15E-04	Tmem86a	TMEM86A	transmembrane protein 86A	Other	other
-2,391	4,76E-04	Usp18	USP18	ubiquitin specific peptidase 18	Cytoplasm	peptidase
-2,390	1,32E-02	Gsdmc3	GSDMC	gasdermin C	Cytoplasm	other
-2,387	1,31E-04	Scoc	SCOC	short coiled-coil protein	Cytoplasm	other
-2,386	1,30E-04	Muc4	MUC4	mucin 4, cell surface associated	Extracellular Space	growth factor
-2,379	1,59E-03	Il18	IL18	interleukin 18 (interferon-gamma-inducing factor)	Extracellular Space	cytokine
-2,378	1,55E-04	Prss12	PRSS12	protease, serine, 12 (neurotrypsin, motopsin)	Extracellular Space	peptidase
-2,365	1,83E-04	Apoc3	APOC3	apolipoprotein C-III	Extracellular Space	transporter
-2,362	6,44E-04	Tcrg-V4	Tcrg-V4	T cell receptor gamma, variable 4	Other	other
-2,354	1,95E-04	Hibch	HIBCH	3-hydroxyisobutyryl-CoA hydrolase	Cytoplasm	enzyme
-2,339	3,33E-04	Gstk1	GSTK1	glutathione S-transferase kappa 1	Cytoplasm	enzyme
-2,310	1,64E-03	Galt	GALT	galactose-1-phosphate uridylyltransferase	Cytoplasm	enzyme
-2,309	3,45E-04	Gstk1	GSTK1	glutathione S-transferase kappa 1	Cytoplasm	enzyme
-2,286	1,08E-04	Scamp5	SCAMP5	secretory carrier membrane protein 5	Cytoplasm	other
-2,279	1,89E-04	Cnnm2	CNNM2	cyclin M2	Plasma Membrane	other
-2,258	2,30E-04	Plcd3	PLCD3	phospholipase C, delta 3	Cytoplasm	enzyme
-2,254	1,81E-04	Muc4	MUC4	mucin 4, cell surface associated	Extracellular Space	growth factor
-2,254	3,42E-04	Rapgef6	RAPGEF6	Rap guanine nucleotide exchange factor (GEF) 6	Plasma Membrane	other
-2,226	1,03E-04	Tm4sf5	TM4SF5	transmembrane 4 L six family member 5	Plasma Membrane	other
-2,220	5,22E-04	Sypl2	SYPL2	synaptophysin-like 2	Other	other
-2,218	1,88E-03	1700055D18Rik	1700055D18Rik	RIKEN cDNA 1700055D18 gene	Other	other
-2,188	3,82E-04	Plekhf1	PLEKHF1	pleckstrin homology domain containing, family F (with FYVE domain) member 1	Cytoplasm	other
-2,181	7,91E-04	Timp1	TIMP1	TIMP metallopeptidase inhibitor 1	Extracellular Space	other
-2,164	2,14E-04	Tha1	Tha1	threonine aldolase 1	Other	other
-2,157	2,50E-03	Nudt5	NUDT5	nudix (nucleoside diphosphate linked moiety X)-type motif 5	Cytoplasm	phosphatase
-2,155	1,71E-03	Cxcl2	CXCL3	chemokine (C-X-C motif) ligand 3	Extracellular Space	cytokine
-2,149	1,22E-03	Slc28a2	SLC28A2	solute carrier family 28 (concentrative nucleoside transporter), member 2	Plasma Membrane	transporter
-2,136	5,76E-03	Hoxa9	HOXA9	homeobox A9	Nucleus	transcription regulator
-2,117	7,39E-04	Unc93a	UNC93A	unc-93 homolog A (C. elegans)	Plasma Membrane	other
-2,115	1,22E-03	Aldh4a1	ALDH4A1	aldehyde dehydrogenase 4 family, member A1	Cytoplasm	enzyme
-2,115	2,54E-04	Slc5a9	SLC5A9	solute carrier family 5 (sodium/sugar cotransporter), member 9	Plasma Membrane	transporter
-2,113	6,53E-03	Snrpb	SNRPB	small nuclear ribonucleoprotein polypeptides B and B1	Nucleus	other
-2,109	1,91E-03	Hvcn1	HVCN1	hydrogen voltage-gated channel 1	Plasma Membrane	ion channel
-2,108	1,38E-03	Nova1	NOVA1	neuro-oncological ventral antigen 1	Nucleus	other
-2,095	1,19E-02	B3galt5	B3GALT5	UDP-Gal:betaGlcNAc beta 1,3-galactosyltransferase, polypeptide 5	Cytoplasm	enzyme
-2,077	1,22E-02	Nbeal1	NBEAL1	neurobeachin-like 1	Other	other
-2,073	1,42E-04	Tmem53	TMEM53	transmembrane protein 53	Other	other
-2,072	4,30E-04	Cyp2d22	Cyp2d22	cytochrome P450, family 2, subfamily d, polypeptide 22	Cytoplasm	enzyme
-2,071	5,56E-03	Olfr1221	Olfr1221	olfactory receptor 1221	Plasma Membrane	G-protein coupled receptor
-2,070	6,22E-04	Hipk2	HIPK2	homeodomain interacting protein kinase 2	Nucleus	kinase
-2,057	3,02E-03	Hdlbp	HDLBP	high density lipoprotein binding protein	Nucleus	transporter
-2,049	2,53E-03	Necab1	NECAB1	N-terminal EF-hand calcium binding protein 1	Cytoplasm	other
-2,048	1,46E-04	Mmp9	MMP9	matrix metallopeptidase 9 (gelatinase B, 92kDa gelatinase, 92kDa type IV collagenase)	Extracellular Space	peptidase
-2,040	3,01E-04	4933422H20Rik	4933422H20Rik	RIKEN cDNA 4933422H20 gene	Other	other
-2,039	2,08E-04	Scoc	SCOC	short coiled-coil protein	Cytoplasm	other
-2,038	1,17E-02	Glra3	GLRA3	glycine receptor, alpha 3	Plasma Membrane	ion channel
-2,028	1,81E-03	Timp1	TIMP1	TIMP metallopeptidase inhibitor 1	Extracellular Space	other
-2,020	2,79E-02	Gsdmc3	GSDMC	gasdermin C	Cytoplasm	other
-2,011	2,16E-04	Slc5a9	SLC5A9	solute carrier family 5 (sodium/sugar cotransporter), member 9	Plasma Membrane	transporter
2,001	5,79E-04	Vpreb2	Vpreb1/Vpreb2	pre-B lymphocyte gene 1	Plasma Membrane	other
2,003	6,28E-03	Bdnf	BDNF	brain-derived neurotrophic factor	Extracellular Space	growth factor
2,004	2,77E-04	Scin	SCIN	scinderin	Cytoplasm	other
2,009	1,07E-02	Gulo	Gulo	gulonolactone (L-) oxidase	Cytoplasm	enzyme
2,009	2,57E-04	P2ry6	P2RY6	pyrimidinergic receptor P2Y, G-protein coupled, 6	Plasma Membrane	G-protein coupled receptor
2,009	4,28E-02	Prf1	PRF1	perforin 1 (pore forming protein)	Cytoplasm	other
2,010	4,86E-04	Mettl7b	METTL7B	methyltransferase like 7B	Other	enzyme
2,013	6,83E-03	Usp13	USP13	ubiquitin specific peptidase 13 (isopeptidase T-3)	Other	peptidase
2,015	4,58E-04	Nudt4	NUDT4	nudix (nucleoside diphosphate linked moiety X)-type motif 4	Cytoplasm	phosphatase
2,017	4,17E-03	Ly6a	Ly6a (includes others)	lymphocyte antigen 6 complex, locus A	Plasma Membrane	other
2,022	4,70E-02	Dr1	DR1	down-regulator of transcription 1, TBP-binding (negative cofactor 2)	Nucleus	transcription regulator
2,022	3,53E-04	Pmvk	PMVK	phosphomevalonate kinase	Cytoplasm	kinase
2,030	3,08E-04	Olfr1178	Olfr1178	olfactory receptor 1178	Plasma Membrane	G-protein coupled receptor
2,032	6,87E-04	Slc11a2	SLC11A2	solute carrier family 11 (proton-coupled divalent metal ion transporter), member 2	Plasma Membrane	transporter
2,036	2,22E-02	Itgb2l	Itgb2l	integrin beta 2-like	Plasma Membrane	other
2,040	7,46E-03	Gcnt2	GCNT2	glucosaminyl (N-acetyl) transferase 2, I-branching enzyme (I blood group)	Cytoplasm	enzyme
2,051	1,74E-04	Cxadr	CXADR	coxsackie virus and adenovirus receptor	Plasma Membrane	transmembrane receptor
2,055	6,23E-04	Mgst1	MGST1	microsomal glutathione S-transferase 1	Cytoplasm	enzyme
2,057	3,76E-02	Clec4g	CLEC4G	C-type lectin domain family 4, member G	Plasma Membrane	other
2,060	6,31E-04	Gls	GLS	glutaminase	Cytoplasm	enzyme
2,062	4,23E-04	Dhcr24	DHCR24	24-dehydrocholesterol reductase	Cytoplasm	enzyme
2,064	1,42E-03	Moap1	MOAP1	modulator of apoptosis 1	Cytoplasm	other
2,073	6,47E-04	Fbxo32	FBXO32	F-box protein 32	Cytoplasm	enzyme
2,080	2,60E-04	Dhcr24	DHCR24	24-dehydrocholesterol reductase	Cytoplasm	enzyme
2,082	3,48E-04	Aacs	AACS	acetoacetyl-CoA synthetase	Cytoplasm	enzyme
2,084	9,92E-03	Olfr67	Olfr67	olfactory receptor 67	Plasma Membrane	G-protein coupled receptor
2,086	1,80E-04	Sult1c2	SULT1C2	sulfotransferase family, cytosolic, 1C, member 2	Cytoplasm	enzyme
2,097	6,29E-04	Cacnb3	CACNB3	calcium channel, voltage-dependent, beta 3 subunit	Plasma Membrane	ion channel
2,097	2,61E-02	Gstcd	GSTCD	glutathione S-transferase, C-terminal domain containing	Other	enzyme
2,098	4,07E-04	Pla2g10	PLA2G10	phospholipase A2, group X	Cytoplasm	enzyme
2,099	3,92E-04	Prmt2	PRMT2	protein arginine methyltransferase 2	Nucleus	enzyme
2,104	4,51E-02	Zxda	Zxda	zinc finger, X-linked, duplicated A	Other	other
2,107	3,71E-02	Taf4a	TAF4	TAF4 RNA polymerase II, TATA box binding protein (TBP)-associated factor, 135kDa	Nucleus	transcription regulator
2,115	4,06E-02	Scfd2	SCFD2	sec1 family domain containing 2	Other	transporter
2,119	4,95E-04	Ptprj	PTPRJ	protein tyrosine phosphatase, receptor type, J	Plasma Membrane	phosphatase
2,123	2,38E-02	Cyp2c37	CYP2C18	cytochrome P450, family 2, subfamily C, polypeptide 18	Cytoplasm	enzyme
2,126	1,90E-03	Olfr25	Olfr25	olfactory receptor 25	Plasma Membrane	G-protein coupled receptor
2,132	1,73E-02	BC021785	BC021785	cDNA sequence BC021785	Cytoplasm	transporter
2,135	2,01E-02	Cdh11	CDH11	cadherin 11, type 2, OB-cadherin (osteoblast)	Plasma Membrane	other
2,135	1,64E-03	Nbl1	MINOS1-NBL1/NBL1	neuroblastoma 1, DAN family BMP antagonist	Nucleus	other
2,137	2,56E-02	Cyp2d9	CYP2D6	cytochrome P450, family 2, subfamily D, polypeptide 6	Cytoplasm	enzyme
2,141	2,59E-04	Aldh8a1	ALDH8A1	aldehyde dehydrogenase 8 family, member A1	Other	enzyme
2,145	3,12E-04	Dsg2	DSG2	desmoglein 2	Plasma Membrane	other
2,150	1,07E-04	Agfg2	AGFG2	ArfGAP with FG repeats 2	Other	other
2,150	2,80E-04	Lss	LSS	lanosterol synthase (2,3-oxidosqualene-lanosterol cyclase)	Cytoplasm	enzyme
2,155	6,04E-04	Tns4	TNS4	tensin 4	Cytoplasm	other
2,157	2,20E-03	Olfr385	Olfr385	olfactory receptor 385	Plasma Membrane	G-protein coupled receptor
2,164	3,75E-03	Ntn4	NTN4	netrin 4	Extracellular Space	other
2,170	1,04E-03	Krba1	KRBA1	KRAB-A domain containing 1	Other	other
2,175	2,11E-03	Spred2	SPRED2	sprouty-related, EVH1 domain containing 2	Extracellular Space	cytokine
2,177	1,08E-03	Gnat1	GNAT1	guanine nucleotide binding protein (G protein), alpha transducing activity polypeptide 1	Plasma Membrane	enzyme
2,180	1,69E-02	4833431D13Rik	4833431D13Rik	RIKEN cDNA 4833431D13 gene	Other	other
2,184	5,06E-03	Gaa	GAA	glucosidase, alpha; acid	Cytoplasm	enzyme
2,189	8,50E-04	Slc25a20	SLC25A20	solute carrier family 25 (carnitine/acylcarnitine translocase), member 20	Cytoplasm	transporter
2,191	3,63E-04	Mapk4	MAPK4	mitogen-activated protein kinase 4	Cytoplasm	kinase

2,193	1,67E-02	Mael	MAEL	maelstrom spermatogenic transposon silencer	Cytoplasm	other
2,193	2,03E-04	Ptprd	Ptprd	protein tyrosine phosphatase, receptor type, D	Plasma Membrane	phosphatase
2,194	4,17E-04	Grip1	GRIP1	glutamate receptor interacting protein 1	Plasma Membrane	transcription regulator
2,198	1,63E-04	Atp2b1	ATP2B1	ATPase, Ca++ transporting, plasma membrane 1	Plasma Membrane	transporter
2,198	3,69E-02	Tpr	TPR	translocated promoter region, nuclear basket protein	Nucleus	transporter
2,200	3,39E-04	Hmgcr	HMGCR	3-hydroxy-3-methylglutaryl-CoA reductase	Cytoplasm	enzyme
2,202	3,88E-04	Ghrl	GHRL	ghrelin/obestatin prepropeptide	Extracellular Space	growth factor
2,210	1,84E-02	Psors1c2	PSORS1C2	psoriasis susceptibility 1 candidate 2	Extracellular Space	other
2,212	1,33E-03	Sycp1-ps1	Sycp1-ps1	synaptonemal complex protein 1, pseudogene 1	Other	other
2,216	3,77E-03	Grk4	GRK4	G protein-coupled receptor kinase 4	Plasma Membrane	kinase
2,224	4,53E-04	Cap2	CAP2	CAP, adenylate cyclase-associated protein, 2 (yeast)	Plasma Membrane	other
2,235	7,27E-04	Tst	TST	thiosulfate sulfurtransferase (rhodanese)	Cytoplasm	enzyme
2,239	1,45E-02	Eif4e1b	EIF4E1B	eukaryotic translation initiation factor 4E family member 1B	Other	other
2,243	1,86E-04	Adk	ADK	adenosine kinase	Nucleus	kinase
2,243	4,73E-04	Mt1	MT1E	metallothionein 1E	Cytoplasm	other
2,247	6,76E-04	Ghrl	GHRL	ghrelin/obestatin prepropeptide	Extracellular Space	growth factor
2,249	3,51E-03	Akr1c19	Akr1c19	aldo-keto reductase family 1, member C19	Other	other
2,255	1,97E-04	Aldoc	ALDOC	aldolase C, fructose-bisphosphate	Cytoplasm	enzyme
2,258	1,68E-02	Tcf12	TCF12	transcription factor 12	Nucleus	transcription regulator
2,268	2,78E-03	4930430A15Rik	4930430A15Rik	RIKEN cDNA 4930430A15 gene	Nucleus	transcription regulator
2,271	2,39E-04	Fbxo32	FBXO32	F-box protein 32	Cytoplasm	enzyme
2,273	5,62E-03	4933403O08Rik	4933403O08Rik	RIKEN cDNA 4933403O08 gene	Other	other
2,280	1,82E-04	Hsd17b7	HSD17B7	hydroxysteroid (17-beta) dehydrogenase 7	Cytoplasm	enzyme
2,283	1,72E-02	F8	F8	coagulation factor VIII, procoagulant component	Extracellular Space	peptidase
2,286	1,36E-02	Olfr382	Olfr382	olfactory receptor 382	Plasma Membrane	G-protein coupled receptor
2,287	5,34E-04	Ugt1a6b	UGT1A6	UDP glucuronosyltransferase 1 family, polypeptide A6	Cytoplasm	enzyme
2,290	1,64E-04	Papss1	PAPSS1	3'-phosphoadenosine 5'-phosphosulfate synthase 1	Cytoplasm	enzyme
2,295	2,35E-04	Plscr1	PLSCR1	phospholipid scramblase 1	Plasma Membrane	enzyme
2,296	5,64E-04	Wdr16	WDR16	WD repeat domain 16	Cytoplasm	other
2,302	1,03E-03	Aldh1a1	ALDH1A1	aldehyde dehydrogenase 1 family, member A1	Cytoplasm	enzyme
2,309	6,40E-03	Guf1	GUF1	GUF1 GTPase homolog (S. cerevisiae)	Cytoplasm	other
2,313	1,07E-04	Tfpi	TFPI	tissue factor pathway inhibitor (lipoprotein-associated coagulation inhibitor)	Extracellular Space	other
2,314	1,39E-02	Taf7	TAF7	TAF7 RNA polymerase II, TATA box binding protein (TBP)-associated factor, 55kDa	Nucleus	transcription regulator
2,324	2,92E-04	Hoxb7	HOXB7	homeobox B7	Nucleus	transcription regulator
2,340	2,46E-04	Prmt2	PRMT2	protein arginine methyltransferase 2	Nucleus	enzyme
2,350	1,27E-04	Tmem65	TMEM65	transmembrane protein 65	Cytoplasm	other
2,354	1,29E-02	Dct	DCT	dopachrome tautomerase	Cytoplasm	enzyme
2,356	7,09E-03	Atg3	ATG3	autophagy related 3	Cytoplasm	enzyme
2,369	1,09E-04	Papss1	PAPSS1	3'-phosphoadenosine 5'-phosphosulfate synthase 1	Cytoplasm	enzyme
2,369	3,76E-02	Serpina3m	Serpina3k (includes others)	serine (or cysteine) peptidase inhibitor, clade A, member 3K	Extracellular Space	other
2,380	6,40E-04	Itpr2	ITPR2	inositol 1,4,5-trisphosphate receptor, type 2	Cytoplasm	ion channel
2,383	1,97E-03	Slc6a20b	Slc6a20b	solute carrier family 6 (neurotransmitter transporter), member 20B	Plasma Membrane	transporter
2,410	1,29E-02	Cpb2	CPB2	carboxypeptidase B2 (plasma)	Extracellular Space	peptidase
2,411	8,02E-03	Camk2a	CAMK2A	calcium/calmodulin-dependent protein kinase II alpha	Cytoplasm	kinase
2,420	2,42E-02	Olfr292	Olfr292 (includes others)	olfactory receptor 297	Plasma Membrane	G-protein coupled receptor
2,427	1,09E-03	Inmt	INMT	indolethylamine N-methyltransferase	Cytoplasm	enzyme
2,460	3,72E-04	Olfr771	Olfr771	olfactory receptor 771	Plasma Membrane	G-protein coupled receptor
2,494	3,39E-02	Ankk1	ANKK1	ankyrin repeat and kinase domain containing 1	Cytoplasm	kinase
2,509	2,13E-04	Ttyh3	TTYH3	tweety family member 3	Plasma Membrane	ion channel
2,510	7,74E-04	Sstr4	SSTR4	somatostatin receptor 4	Plasma Membrane	G-protein coupled receptor
2,512	4,72E-02	Ablim1	ABLIM1	actin binding LIM protein 1	Cytoplasm	other
2,518	1,32E-03	Fa2h	FA2H	fatty acid 2-hydroxylase	Cytoplasm	enzyme
2,519	1,47E-04	Fbxo32	FBXO32	F-box protein 32	Cytoplasm	enzyme
2,537	3,92E-04	Timd2	Timd2	T cell immunoglobulin and mucin domain containing 2	Plasma Membrane	other
2,543	7,77E-03	Cpb2	CPB2	carboxypeptidase B2 (plasma)	Extracellular Space	peptidase
2,564	3,45E-02	Cyp2c70	Cyp2c70	cytochrome P450, family 2, subfamily c, polypeptide 70	Cytoplasm	enzyme
2,579	2,27E-02	Ern1	ERN1	endoplasmic reticulum to nucleus signaling 1	Cytoplasm	kinase
2,581	3,60E-02	Serpina10	SERPINA10	serpin peptidase inhibitor, clade A (alpha-1 antiproteinase, antitrypsin), member 10	Extracellular Space	other
2,588	9,17E-04	Mpst	MPST	mercaptopyruvate sulfurtransferase	Cytoplasm	enzyme
2,604	1,32E-04	Pmp22	PMP22	peripheral myelin protein 22	Plasma Membrane	other
2,634	1,44E-02	Slco1b2	SLCO1B3	solute carrier organic anion transporter family, member 1B3	Plasma Membrane	transporter
2,653	1,92E-04	Krt84	KRT84	keratin 84	Cytoplasm	other
2,654	1,38E-04	Xpnpep2	XPNPEP2	X-prolyl aminopeptidase (aminopeptidase P) 2, membrane-bound	Plasma Membrane	peptidase
2,678	2,15E-04	Ugt2b36	UGT2B7	UDP glucuronosyltransferase 2 family, polypeptide B7	Cytoplasm	enzyme
2,686	1,54E-03	Olfr126	Olfr126 (includes others)	olfactory receptor 126	Plasma Membrane	G-protein coupled receptor
2,696	4,76E-02	Itih4	ITIH4	inter-alpha-trypsin inhibitor heavy chain family, member 4	Extracellular Space	other
2,701	1,11E-02	Tbx15	TBX15	T-box 15	Nucleus	transcription regulator
2,736	9,84E-05	Tceal8	TCEAL8	transcription elongation factor A (SII)-like 8	Other	other
2,742	5,62E-05	Ptprd	Ptprd	protein tyrosine phosphatase, receptor type, D	Plasma Membrane	phosphatase
2,764	5,39E-04	Fhl1	FHL1	four and a half LIM domains 1	Cytoplasm	other
2,765	1,99E-04	Nsdhl	NSDHL	NAD(P) dependent steroid dehydrogenase-like	Cytoplasm	enzyme
2,771	5,05E-05	Ocm	OCM	oncomodulin	Cytoplasm	other
2,800	7,70E-05	Pibf1	PIBF1	progesterone immunomodulatory binding factor 1	Nucleus	other
2,807	1,82E-02	Veph1	VEPH1	ventricular zone expressed PH domain-containing 1	Nucleus	other
2,814	3,31E-04	Cpn1	CPN1	carboxypeptidase N, polypeptide 1	Extracellular Space	peptidase
2,814	3,72E-05	Ptprd	Ptprd	protein tyrosine phosphatase, receptor type, D	Plasma Membrane	phosphatase
2,835	4,57E-04	3300005D01Rik	3300005D01Rik	RIKEN cDNA 3300005D01 gene	Other	other
2,867	4,45E-04	Fhl1	FHL1	four and a half LIM domains 1	Cytoplasm	other
2,869	1,19E-04	Nsdhl	NSDHL	NAD(P) dependent steroid dehydrogenase-like	Cytoplasm	enzyme
2,877	1,46E-02	Cyp2e1	CYP2E1	cytochrome P450, family 2, subfamily E, polypeptide 1	Cytoplasm	enzyme
2,880	1,50E-04	Hoxb7	HOXB7	homeobox B7	Nucleus	transcription regulator
2,901	1,58E-04	Nsdhl	NSDHL	NAD(P) dependent steroid dehydrogenase-like	Cytoplasm	enzyme
2,907	1,82E-02	Itih1	ITIH1	inter-alpha-trypsin inhibitor heavy chain 1	Extracellular Space	other
2,924	1,19E-04	Xpnpep2	XPNPEP2	X-prolyl aminopeptidase (aminopeptidase P) 2, membrane-bound	Plasma Membrane	peptidase
2,954	5,81E-05	9130024F11Rik	9130024F11Rik	RIKEN cDNA 9130024F11 gene	Other	other
2,973	7,95E-05	Plscr1	PLSCR1	phospholipid scramblase 1	Plasma Membrane	enzyme
3,016	3,91E-03	Mtmr6	MTMR6	myotubularin related protein 6	Cytoplasm	phosphatase
3,109	9,38E-04	Cyp2c55	CYP2C9	cytochrome P450, family 2, subfamily C, polypeptide 9	Cytoplasm	enzyme
3,189	1,73E-04	Epb4.1l4a	EPB41L4A	erythrocyte membrane protein band 4.1 like 4A	Extracellular Space	other
3,205	7,38E-05	Slc13a2	SLC13A2	solute carrier family 13 (sodium-dependent dicarboxylate transporter), member 2	Plasma Membrane	transporter
3,208	3,34E-05	Fgfbp1	FGFBP1	fibroblast growth factor binding protein 1	Extracellular Space	other
3,217	1,28E-04	Ugt2b36	UGT2B7	UDP glucuronosyltransferase 2 family, polypeptide B7	Cytoplasm	enzyme
3,220	1,14E-04	Rasd2	RASD2	RASD family, member 2	Cytoplasm	enzyme
3,230	4,61E-05	Cyp51	CYP51A1	cytochrome P450, family 51, subfamily A, polypeptide 1	Cytoplasm	enzyme
3,257	4,42E-05	Cables1	CABLES1	Cdk5 and Abl enzyme substrate 1	Nucleus	other
3,310	5,48E-03	Ttr	TTR	transthyretin	Extracellular Space	transporter
3,336	8,52E-05	4732465J04Rik	4732465J04Rik	RIKEN cDNA 4732465J04 gene	Other	other
3,379	9,40E-05	Abi3bp	ABI3BP	ABI family, member 3 (NESH) binding protein	Extracellular Space	other
3,423	2,58E-04	Timd2	Timd2	T cell immunoglobulin and mucin domain containing 2	Plasma Membrane	other
3,437	3,56E-03	Ugt1a2	UGT1A3	UDP glucuronosyltransferase 1 family, polypeptide A3	Cytoplasm	enzyme
3,569	4,18E-02	Mup3	Mup1 (includes others)	major urinary protein 1	Extracellular Space	other
3,573	8,08E-05	Bex4	BEX4	brain expressed, X-linked 4	Cytoplasm	other
3,587	3,58E-02	Itih4	ITIH4	inter-alpha-trypsin inhibitor heavy chain family, member 4	Extracellular Space	other
3,591	9,72E-05	Pcsk9	PCSK9	proprotein convertase subtilisin/kexin type 9	Extracellular Space	peptidase
3,628	1,41E-04	Eif4ebp3	EIF4EBP3	eukaryotic translation initiation factor 4E binding protein 3	Cytoplasm	other
3,640	2,82E-02	Kng1	KNG1	kininogen 1	Extracellular Space	other
3,929	4,36E-02	Fga	FGA	fibrinogen alpha chain	Extracellular Space	other
3,946	5,36E-05	Slc13a1	SLC13A1	solute carrier family 13 (sodium/sulfate symporter), member 1	Plasma Membrane	transporter
4,063	3,79E-05	Lamb3	LAMB3	laminin, beta 3	Extracellular Space	transporter
4,065	4,54E-05	Pmp22	PMP22	peripheral myelin protein 22	Plasma Membrane	other
4,353	4,15E-02	Pnlip	PNLIP	pancreatic lipase	Extracellular Space	enzyme
4,355	2,59E-05	Zdhhc19	ZDHHC19	zinc finger, DHHC-type containing 19	Other	other
4,493	4,13E-02	Hpx	HPX	hemopexin	Extracellular Space	transporter
4,620	3,75E-02	Mup2	Mup1 (includes others)	major urinary protein 1	Extracellular Space	other
4,935	1,34E-04	Hmgcs2	HMGCS2	3-hydroxy-3-methylglutaryl-CoA synthase 2 (mitochondrial)	Cytoplasm	enzyme
5,033	1,40E-03	Cfl2	CFL2	cofilin 2 (muscle)	Other	other
5,067	4,04E-05	Hspb1	HSPB1	heat shock 27kDa protein 1	Cytoplasm	other
5,338	3,60E-05	Pik3ap1	PIK3AP1	phosphoinositide-3-kinase adaptor protein 1	Cytoplasm	other
5,361	6,81E-05	Gsta3	GSTA3	glutathione S-transferase alpha 3	Cytoplasm	enzyme
5,406	3,53E-05	Bex4	BEX4	brain expressed, X-linked 4	Cytoplasm	other
5,445	4,93E-03	Sycn	SYCN	syncollin	Extracellular Space	other
6,319	1,83E-05	Gcnt1	GCNT1	glucosaminyl (N-acetyl) transferase 1, core 2	Cytoplasm	enzyme
6,544	4,42E-04	Serpina1b	SERPINA1	serpin peptidase inhibitor, clade A (alpha-1 antiproteinase, antitrypsin), member 1	Extracellular Space	other
6,632	5,88E-04	Serpina1d	SERPINA1	serpin peptidase inhibitor, clade A (alpha-1 antiproteinase, antitrypsin), member 1	Extracellular Space	other
6,893	1,45E-05	Gcnt1	GCNT1	glucosaminyl (N-acetyl) transferase 1, core 2	Cytoplasm	enzyme
7,573	3,13E-02	Sertad4	SERTAD4	SERTA domain containing 4	Other	other
7,755	2,40E-04	Serpina1b	SERPINA1	serpin peptidase inhibitor, clade A (alpha-1 antiproteinase, antitrypsin), member 1	Extracellular Space	other
7,870	1,85E-04	Serpina1b	SERPINA1	serpin peptidase inhibitor, clade A (alpha-1 antiproteinase, antitrypsin), member 1	Extracellular Space	other
7,983	2,21E-05	Cubn	CUBN	cubilin (intrinsic factor-cobalamin receptor)	Plasma Membrane	transmembrane receptor
9,371	6,31E-06	Slc40a1	SLC40A1	solute carrier family 40 (iron-regulated transporter), member 1	Plasma Membrane	transporter
12,336	6,94E-06	Defb37	Defb37	defensin beta 37	Extracellular Space	other

Table S4. Primers used for qPCR analysis of gene transcripts

Claudin-1
Up 5'-GCCATCTACGAGGGACTGTGGA-3'
Dw 5'-TGCCAATTACCATCAAGGCTCGG-3'

Claudin-2
Up 5'-GCCACCCACAGATACTTGTAAGGAG-3'
Dw 5'-TTCGGAGCCTGTTTGCTTGTCG-3'

Claudin-3
Up 5'-CCTTGCTGTGTTGCTCCTGCC-3'
Dw 5'-CTCAGACGTAGTCCTTGCGGTC-3'

ZO-1 (TJP1)
Up 5'-TGCAGAGACAATAGCATTCTCCCAC-3'
Dw 5'-TAGCTCCACGGGCTTCAGGAAC-3'

Jam-1 (F11R)
Up 5'-TGGATATGGGACAGCCATGAGGT-3'
Dw 5'-GCTATAGGCAAACCAGACGCCA-3'

Occludin
Up 5'-GGAACACATTTATGATGAACAGCCCCC-3'
Dw 5'-CGCTTGCCATTCACTTTGCCAT-3'

E-cadherin
Up 5'-ACCAAAGTGACGCTGAAGTCCAT-3'
Dw 5'-GATGGGAGGGATGACCCAGTCT-3'

Icam1
Up 5'-CTGCTACCTGCACTTTGCCCTG-3'
Dw 5'-AGGCTTCTCTGGGATGGATGGAT-3'

Cx3cr1
Up 5'-GAGTATGACGATTCTGCTGAGG-3'
Dw 5'-CAGACCGAACGTGAAGACGAG-3'

Il-1β
Up 5'-TAGCCCGCACTGAGGTCTTT-3'
Dw 5'-AGCAATGTGCTGGTGCTTCA-3'

Cd5
Up 5'-CCCCACAGGAGTGAACCAGAACA-3'
Dw 5'-TGGATAAGCAGACAGGTGAGAGTTCC-3'

Cd69
Up 5'-ACTTCTTCTCCACCACAACCAAGAGT-3'
Dw 5'-GCCCAATCCAATGTTCCAGTTCACC-3'

Il-1βr
Up 5'-TCGCTATCCGTTTATCTGTGTTGTTAAGAA-3'
Dw 5'-TGTAGCCGTGAGGATGATAAAGCCC-3'

Il-23a

Up	5’-CCCGTATCCAGTGTGAAGATGGTTGT-3’	

Up 5’-CCCGTATCCAGTGTGAAGATGGTTGT-3’
Dw 5’-TCCCCTTTGAAGATGTCAGAGTCAAGC-3’

Ccl20

Up 5’-GCCTCTCGTACATACAGACGC-3’
Dw 5’-CCAGTTCTGCTTTGGATCAGC-3’

Ccr6

Up 5’-CCTGGGCAACATTATGGTGGT-3’
Dw 5’-CAGAACGGTAGGGTGAGGACA-3’

Cxcl1

Up 5’-CTGGGATTCACCTCAAGAACATC-3’
Dw 5’-CAGGGTCAAGGCAAGCCTC-3’

Il-21

Up 5’-GGACCCTTGTCTGTCTGGTAG-3’
Dw 5’-TGTGGAGCTGATAGAAGTTCAGG-3’

Ccr4

Up 5’-TCCCCTCGTGCTCTGCA-3’
Dw 5’-TGCGTGTAAGAGGAGCTGGAC-3’

Cd25

Up 5’-TTGTCGGGCAGAACTGTGTCT-3’
Dw 5’-GCTCCAGGAGTTTCCTAAGCAA-3’

Cxcr4

Up 5’-AGCATGACGGACAAGTACC-3’
Dw 5’-GATGATATGGACAGCCTTACAC-3’

Foxp3

Up 5’-GGCCCTTCTCCAGGACAGA-3’
Dw 5’-GCTGATCATGGCTGGGTTGT-3’

Ifn-γ

Up 5’-CAGCAACAGCAAGGCGAAA-3’
Dw 5’-GCTGGATTCCGGCAACAG-3’

Il-10

Up 5’-TTTGAATTCCCTGGGTGAGAAG-3’
Dw 5’-TGCTCCACTGCCTTGCTCTT-3’

Il-12p35

Up 5’-CACGCTACCTCCTCTTTTTGG-3’
Dw 5’-TTTTCTCTGGCCGTCTTCAC-3’

Il-12p40

Up 5’-GGTGCAAAGAAACATGGACTTG-3’
Dw 5’-GACAGAGACGCCATTCCACAT-3’

Il-13

Up 5’-GTGTCTCTCCCTCTGACCCTTAA-3’
Dw 5’-GAGATGTTGGTCAGGGAATCC-3’

Il-17

	Up	5’-TCCAGAAGGCCCTCAGACTAC-3’
	Dw	5’-TGTGGTGGTCCAGCTTTCC-3’

Il-2

	Up	5’-CCCAGGATGCTCACCTTCAA-3’
	Dw	5’-CCGCAGAGGTCCAAGTTCAT-3’

Il-23r

	Up	5’-GCCAAGAAGACCATTCCCGA-3’
	Dw	5’-TCAGTGCTACAATCTTCTTCAGAGGACA-3’

Il-24

	Up	5’-CTCCACTCTGGCCAACAACTT-3’
	Dw	5’-CACCAAAGCGACTTCTGTATCC-3’

Tgfβ

	Up	5’-GGACCCTGCCCCTATATTTG-3’
	Dw	5’-GCTTGCGACCCACGTAGTAG-3’

Tnfα

	Up	5’-AGGCGGTGCCTATGTCTCA-3’
	Dw	5’-GGGTCTGGGCCATAGAACTG-3’

Table S5. Primers used for qPCR analysis of ChIPs experiments

Site 1

mClau2prom-331up1	5’-TGACATTTTGGCTCTACTTCCA-3’
mClau2prom-157dw1	5’CAGGCTCAAGAAGGCATCTA-3’

Site 2

mClau2prom-1706up3	5’-ATTACCCCAGGGATGTTGGT-3’
mClau2prom-1528dw3	5’-GGAGGGGGTTAGGATTTAGC-3’

Site 3 (-1775 to -1762)

mClau2prom-18186up5	5’-TGGTGGAAATGAGAAACACAA-3’
mClau2prom-1707dw5	5’-CCTTGACACCCTGTGCCTAT-3’

Negative region

mouseIL1β(-398)-FWD	5’-CCAAACTCCAACTGCAAAGC-3’
mouseIL1β(-197)-REV	5’-TGGTGGAAATGGGCATTATT-3’

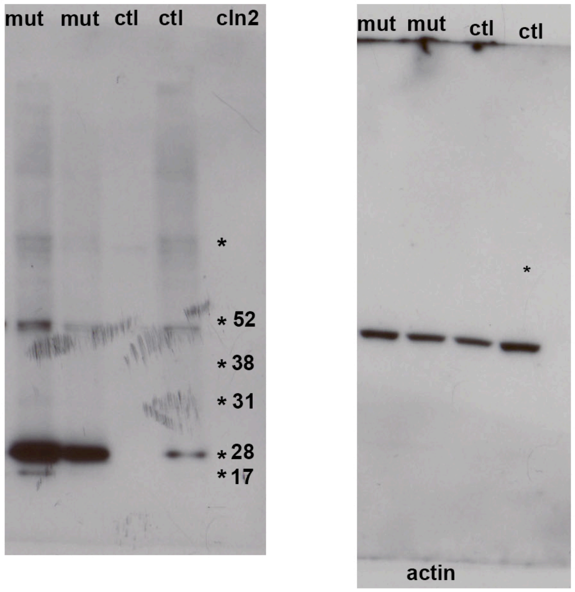

Full length gels for claudin-2 and actin blots displayed in Figure 2b.
